# Evaluating the performance of ChatGPT in patient consultation and image-based preliminary diagnosis in thyroid eye disease

**DOI:** 10.3389/fmed.2025.1546706

**Published:** 2025-02-18

**Authors:** Yue Wang, Shuo Yang, Chengcheng Zeng, Yingwei Xie, Ya Shen, Jian Li, Xiao Huang, Ruili Wei, Yuqing Chen

**Affiliations:** ^1^Department of Ophthalmology, Changzheng Hospital of Naval Medical University, Shanghai, China; ^2^Department of Urology, Beijing Tongren Hospital of Capital Medical University, Beijing, China

**Keywords:** thyroid eye disease, large language model, ChatGPT, virtual healthcare, clinical practice

## Abstract

**Background:**

The emergence of Large Language Model (LLM) chatbots, such as ChatGPT, has great promise for enhancing healthcare practice. Online consultation, accurate pre-diagnosis, and clinical efforts are of fundamental importance for the patient-oriented management system.

**Objective:**

This cross-sectional study aims to evaluate the performance of ChatGPT in inquiries across ophthalmic domains and to focus on Thyroid Eye Disease (TED) consultation and image-based preliminary diagnosis in a non-English language.

**Methods:**

We obtained frequently consulted clinical inquiries from a published reference based on patient consultation data, titled *A Comprehensive Collection of Thyroid Eye Disease Knowledge*. Additionally, we collected facial and Computed Tomography (CT) images from 16 patients with a definitive diagnosis of TED. From 18 to 30 May 2024, inquiries about the TED consultation and preliminary diagnosis were posed to ChatGPT using a new chat for each question. Responses to questions from ChatGPT-4, 4o, and an experienced ocular professor were compiled into three questionnaires, which were evaluated by patients and ophthalmologists on four dimensions: accuracy, comprehensiveness, conciseness, and satisfaction. The preliminary diagnosis of TED was deemed accurate, and the differences in the accuracy rates were further calculated.

**Results:**

For common TED consultation questions, ChatGPT-4o delivered more accurate information with logical consistency, adhering to a structured format of disease definition, detailed sections, and summarized conclusions. Notably, the answers generated by ChatGPT-4o were rated higher than those of ChatGPT-4 and the professor, with accuracy (4.33 [0.69]), comprehensiveness (4.17 [0.75]), conciseness (4.12 [0.77]), and satisfaction (4.28 [0.70]). The characteristics of the evaluators, the response variables, and other quality scores were all correlated with overall satisfaction levels. Based on several facial images, ChatGPT-4 twice failed to make diagnoses because of lacking characteristic symptoms or a complete medical history, whereas ChatGPT-4o accurately identified the pathologic conditions in 31.25% of cases (95% confidence interval, CI: 11.02–58.66%). Furthermore, in combination with CT images, ChatGPT-4o performed comparably to the professor in terms of diagnosis accuracy (87.5, 95% CI 61.65–98.45%).

**Conclusion:**

ChatGPT-4o excelled in comprehensive and satisfactory patient consultation and imaging interpretation, indicating the potential to improve clinical practice efficiency. However, limitations in disinformation management and legal permissions remain major concerns, which require further investigation in clinical practice.

## 1 Introduction

Equipped with extensive medical information and built-in mechanisms for self-checking (1), Large Language Model (LLM) chatbots represent notable advancements in artificial intelligence (AI), acting as one of the advanced technologies for practical tools in virtual healthcare (2). ChatGPT-4 (released on 14 March 2023) has demonstrated human-level proficiency across a range of professional and academic benchmarks, particularly in terms of factual accuracy and adaptability. A subsequent iteration, ChatGPT version 4o (“o” for “omni”), released on 13 May 2024, mainly pre-trained by publicly available data such as diverse web articles and videos, further improves the naturalness of human-computer interaction, particularly in non-English languages, enabling its broader global application (“GPT-4o System Card,” (3, 35).

Thyroid Eye Disease (TED), also known as Thyroid-associated Ophthalmopathy or Graves’ Ophthalmopathy, is a chronic and debilitating autoimmune disorder characterized by infiltrative and proliferative lesions in the retrobulbar and periorbital tissues (4, 5). Because some clinicians are unfamiliar with TED, the condition is frequently misdiagnosed, resulting in delayed or inappropriate treatment (6). Significant changes in appearance, such as severe eyelid retraction, pronounced exophthalmos, and corneal ulceration, have profound negative effects on patients’ work and daily lives, often leading to psychological distress, feelings of inferiority, and reduced social interactions (7, 8).

Importantly, with the aim of promoting public health, almost all hospital departments provide patients with educational materials, allowing them to access essential health information. However, patients tend to search for disease-related topics, focusing mainly on new or inappropriate therapies that can induce unrealistic expectations toward outcomes and potential disputes between clinicians and patients (9). Several studies have explored the role of ChatGPT in the medical field, with promising results in clinical domains. LLM chatbots can also assist in preliminary assessments based on patients’ reported symptoms in a multilingual global environment (10), particularly in remote regions where specialized ophthalmologists are scarce (2, 11).

A key gap in existing research is the lack of patient feedback on the quality of information generated by LLM chatbots. This study seeks to address that gap by involving patients with TED and subspecialty ophthalmologists to evaluate the quality of the responses generated in terms of accuracy, comprehensiveness, conciseness, and overall satisfaction. In this cross-sectional study, the objective of the study was to evaluate the performance of ChatGPT-4 and 4o in patient consultation from multiple dimensions and further explored whether ChatGPT could perform preliminary diagnoses based on images, serving as a valuable supplementary tool in clinical practice.

## 2 Methods

### 2.1 Questions and image source

The common consultation questions in this study were sourced from a printed patient education book, edited by R.L.W., titled *A Comprehensive Collection of Thyroid Eye Disease Knowledge*, which contains 141 commonly asked questions related to TED, focusing on disease treatment and rehabilitation (12). The questions were selected based on more than 40 years of clinical experience, relevance, and clarity from a popular online consultation platform, Haodafu Consultant App (Beijing Xinyi Qiangguo Technology Co.). While excluding several typical prognosis reports, we only included the most straightforward questions for each type with similar meanings. After screening, 15 questions were finalized, covering a broad range of topics such as pathological mechanisms and treatment for TED. Answers to descriptive and binary questions contained elaborate explanations, so we identified the content categories (basic knowledge, diagnosis, mechanism, surgical, and non-surgical treatment, with three questions from each category) and difficulty levels (low, moderate, and high difficulty, with five questions from each category, in Supplementary eTable 1). In the image-based evaluation, 16 cases with a definitive diagnosis of TED were from the Shanghai Changzheng Hospital medical record system, each including several frontal and lateral facial appearance images and CT images from horizontal and coronal positions (detailed in Supplementary eTable 2).

### 2.2 Ethics approval

Ethical approval for this cross-sectional study was obtained from the Ethics Committee of the Changzheng Hospital Biomedical Research Ethics Committee (*Identifier: 2021SL044*). This research adhered to the principles outlined in the Declaration of Helsinki. Due to the utilization of de-identified images and personal information, the requirement for informed consent was waived.

### 2.3 Responses from ChatGPT

In response to the consultation questions, two versions of ChatGPT (Versions 4 and 4o, Open AI) were used from 18 May to 20 May 2024. Under inquiries, “Hi, ChatGPT. I am a patient with TED.” comment was posted. Following the common questions for patient consultation in Chinese, GPT-4 and GPT-4o generated responses for the 15 questions (Supplementary eTable 3, translated by Grammarly software). All identifying elements were removed, including special characters, website links, greetings, etc. The objective characteristics, the number of characters, paragraphs, segments, and photographs in the responses were collected.

When conducting the image-based evaluation, either facial personalized features below orbits or personal information had been mosaic processed, then ChatGPT-4 and 4o were used from 22 May to 30 May 2024. Under inquiries, “Hi, ChatGPT. Assuming as a certified clinician, please walk me through image-based diagnosis and list three in order of likelihood.” was posted in Chinese. Following images from patients with TED, ChatGPT (versions 4 and 4o) performed preliminary diagnoses for the 16 cases. The first section of the preliminary diagnosis was based on several facial images, followed by the section of several facial images combined with CT images (Supplementary eTable 2). In particular, every question was asked using a new chat to ensure consistency and avoid self-learning from the chat history in this study.

### 2.4 Response from the professor

Professor R.L.W’s responses to 15 consultation questions were sourced from the publication he edited, *A Comprehensive Collection of Thyroid Eye Disease Knowledge* (Supplementary eTable 3, translated by Grammarly software). Objective characteristics, the number of characters, paragraphs, segments, and photographs in the answers were also collected. After observing the PowerPoint presentation that displayed facial images of a patient on one page and CT images of the same patients on the following page, the professor performed the preliminary diagnosis.

### 2.5 Quality evaluation of responses

The quality evaluation of responses to the consultation questions was performed as a questionnaire investigation. To enhance reliability, three questionnaires were designed to avoid simultaneously presenting the answers of three generators to the same question to the evaluators and following bias. The three questionnaires each contained the same questions about TED but with different corresponding answers, presented in a consistent and specific order. In other words, since the structure of the questionnaires remained uniform, the answers varied, featuring five responses from ChatGPT-4, five from ChatGPT-4o, and five from an ocular professor’s written publication, arranged randomly.

With the full informed consent of the patients and their accompanying persons, the paper questionnaires were distributed to patients visiting the consulting room and professional ophthalmologists (Department of Ophthalmology, Shanghai Changzheng Hospital). In addition to the instructions from the residents of the ophthalmologist face to face, there was a brief description of the essential matters at the beginning and some questions to collect the evaluator’s sex, age, disease course, duration, treatment experience, and level of knowledge of the disease in the patient questionnaires. In ophthalmologist questionnaires, we collected the evaluator’s sex, age, and professional titles using Likert scales. Despite 15 questions and their answers followed by four quality scores, there is an optional section to collect patient feedback (detailed in Supplementary eTable 4).

The voluntary patients in the questionnaire investigation were all literate, aged between 18 and 60 years old, with at least a preliminary understanding of the TED and the courses of personal diseases. The professional participants were subspecialists in ophthalmology, with clinical experience assisting to diagnose and independently treating patients with TED for more than 2 years. Under specific guidance, all participants scored in a quiet environment without a time limit.

**Figure 1:**
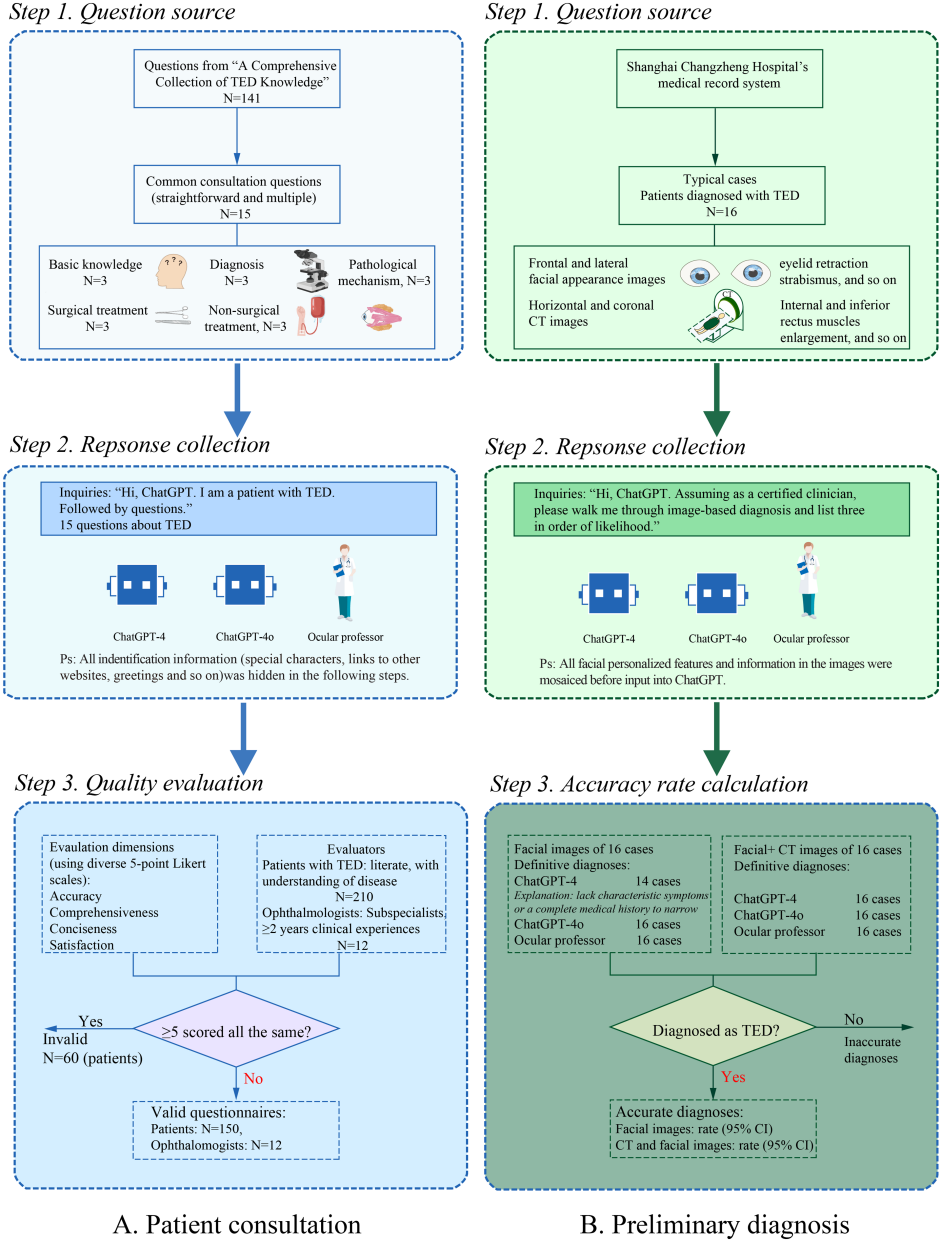
Flowchart of the data extraction process for quality evaluation about TED. **(A)** Patient consultation in 15 clinical questions about TED. **(B)** Image-based preliminary diagnosis using facial and CT images.

Concise and easy-to-understand materials are in great need for patients with poor disease knowledge. Therefore, evaluation tools were designed as predefined 5-point Likert scales, including common quality indicators (accuracy, comprehensiveness), conciseness, and overall satisfaction. The screening criteria were as follows: The questionnaires were considered invalid if at least one-third (five or more questions) scored the same on all four dimensions. Finally, 162 valid questionnaires from 12 ophthalmologists and 150 patients with TED were included (Figure 1A). When evaluating the performance of the image-based preliminary diagnosis, we excluded two non-definitive diagnoses from ChatGPT-4 and then calculated the accuracy rate of image interpretation to diagnose TED (Figure 1B).

### 2.6 Statistical analysis

The quality of responses to various difficulty and content questions about TED was compared in four dimensions: accuracy, comprehensiveness, conciseness, and satisfaction, using the Kruskal-Wallis test. We calculated the correlations between the characteristics of the evaluator, the quality evaluation scores, and the overall satisfaction scores using the Spearman’s correlation analysis. In addition, the independent samples *t*-test (Mann–Whitney test and Welch’s *t*-test) was used to compare quality scores between sex and disease courses using GraphPad Prism (version 10.2.0, GraphPad Software, Inc.). With numerous participants, the reliability or consistency of the questionnaire was verified using Cronbach’s alpha coefficient, and its validity was graded using the Kaizer–Meyer–Olkin and Bartlett sphere tests. The accuracy rate (95% confidence interval, CI) was calculated using Clopper-Pearson and the inter-group differences using the McNemar Chi-square test (SPSS Statistics, version 21.0, IBM). Descriptive analysis, such as median (interquartile range, IQR), mean (standard deviation, SD), and range, was used as appropriate in the manuscript. *p* < 0.05 was considered statistically significant.

## 3 Results

### 3.1 Performance of ChatGPT in TED patient consultation

In combination with responses from ChatGPT-4 and 4o under inquiries to 15 consultation questions, responses generated by an ocular professor (R.L.W.) were collected (total responses for comparison: *N* = 45). The quality variables ordered in three questionnaires were listed as 45 groups to evaluate reliability and validity. Cronbach’s alpha coefficient was 0.831, the Kaiser-Meyer-Olkin value was 0.778, the approximate chi-square value of Bartlett’s sphere test was 2649.992, the degree of freedom was 990, and the *p* value was 0.000, indicating good reliability of scales in questionnaires.

With joint efforts, the questionnaire investigation included 9,720 assessment items and 936 personal information items. The main participants in the primary group were young and middle-aged women in the stable stage of TED who had moderate or higher knowledge levels and experiences of thyroid, intravenous glucocorticoid, or orbit surgeries (Table 1). All professional participants were junior to senior ophthalmologists at Shanghai Changzheng Hospital (Table 2).

**Table 1:** Personal information on patients participating in the questionnaire survey on consultation (*N* = 150).

Group		Number (Percentage)
Gender		
	Male	38 (25.3%)
	Female	112 (74.7%)
Ages (years)		
	18–20	6 (4%)
	21–30	35 (23.3%)
	31–40	47 (31.3%)
	41–50	38 (25.3%)
	51–60	24 (16%)
Disease stage		
	Active	36 (24%)
	Stable	114 (76%)
Disease course		
	<6 months	6 (4%)
	Half-1 year	27 (18%)
	1–3 years	63 (42%)
	3–5 years	28 (18.7%)
	> 5 years	26 (17.3%)
Treatment experiences^a^		
	Treatment on thyroid	110 (73.3%)
	Glucocorticoid	63 (42%)
	Radiotherapy	17 (11.3%)
	Monoclonal antibody	14 (9.3%)
	Orbital surgery	53 (35.3%)
TED knowledge		
	Poor	5 (3.3%)
	Inferior	24 (16%)
	Moderate	55 (36.7%)
	Basic	53 (35.3%)
	Excellent	13 (8.7%)

a Patients with TED may have experienced various treatments during the chronic disease course. Therefore, the total number of five different treatment experiences is more than 150, as the total percentage is more than 100%.

**Table 2:** Personal information on ophthalmologists participating in the questionnaire survey on consultation (*N* = 12).

Group		Number (Percentage)
Gender		
	Male	6 (50%)
	Female	6 (50%)
Age (years)		
	18–20	0 (0%)
	21–30	8 (66.7%)
	31–40	3 (25%)
	41–50	1 (8.3%)
	51–60	0 (0%)
Professional titles		
	Residents	6 (50%)
	Attendings	5 (41.7%)
	Associate professor	1 (8.3%)

The median (IQR) character count was 373 (274–4,449), while that of paragraphs, sections, and photographs was 5 (3–8), 3 (0–5), and 0 (0–0), respectively (Table 3). With hundreds of characters, separating the content into sections was more logical and beneficial to grasp key points. Since most of the answers generated by chatbots (version 4: 66.7%, 4o: 93.3%) remained a uniform structure, the mechanisms, advantages, and disadvantages of the clinical options were explained point by point. For example, in Question 6, interpreting the thyroid function checklist, ChatGPT-4o segmentally summarized three interpretation steps and gave examples of how indicators changed in hyperthyroidism and hypothyroidism, while the professor introduced their synthesis pathways and clinical meanings.

**Table 3:** The reactions of LLM chatbots and an experienced ocular professor to the 15 chosen thyroid eye disease inquiries.

Answer variables	ChatGPT-4	ChatGPT-4o	Ocular professor	Total	*p* value^a^
Characters Median (IQR)	313 (258–377)	404 (373–644)	360 (169–1,017)	373 (274–449)	0.049*
Paragraphs Median (IQR)	5 (3–6)	7 (5–10)	3 (1–9)	5 (3–8)	0.0045**
Sections Median (IQR)	3 (0–4)	5 (3–9)	0 (0–0)	3 (0–5)	0.0008***
Photographs Median (IQR)	0 (0–0)	0 (0–0)	0 (0–1)	0 (0–0)	0.0138*

^aThe p value was described as the Kruskal-Wallis test p value.^

* refers to the *p* < 0.05, ** refers to the *p* < 0.01, and *** refers to the *p* < 0.001.

There were some inappropriate translations of terms in ChatGPT-4, which had been correctly updated in version 4o. In comparison with answers from ChatGPT-4, those of version 4o contained significantly more characters (*p* = 0.045), and the same happened in the paragraphs and sections (*p* = 0.0034 and *p* = 0.0005, respectively) between ChatGPT-4o and the professor (Table 4). In long text, visual impacts such as bold font and schematic diagrams are prominent to attract viewers’ attention and improve understandability. Without special instruction, chatbots did not generate photographs in responses but highlighted key points in bold font at the front of each section. In the professor’s answers, with several clear images, all paragraphs had first-line indentation without highlighting, in line with Chinese language habits.

**Table 4:** The performance of the LLM chatbots and an experienced ocular professor on different difficulty levels and content focuses.

Categories	Quality evaluation	ChatGPT-4	ChatGPT-4o	Professor	*p* value^a^
Low difficulty	Comprehensiveness	4.04 (0.82)	4.23 (0.73)	4.07 (0.79)	0.0126*
	Conciseness	4.13 (0.72)	4.18 (0.77)	4.05 (0.72)	0.0859
Moderate	Comprehensiveness	4.03 (0.72)	4.01 (0.78)	4.13 (0.86)	0.0387*
	Conciseness	4.19 (0.70)	4.03 (0.80)	4.01 (0.84)	0.0460*
High difficulty	Comprehensiveness	3.98 (0.78)	4.27 (0.71)	4.11 (0.70)	<0.0001****
	Conciseness	4.07 (0.78)	4.14 (0.74)	3.94 (0.79)	0.0160*
Diagnosis	Accuracy	4.25 (0.78)	4.49 (0.62)	4.35 (0.69)	0.0162*
	Comprehensiveness	4.02 (0.74)	4.27 (0.67)	4.26 (0.74)	0.0022**
	Conciseness	4.13 (0.74)	4.10 (0.82)	3.72 (0.81)	<0.0001****
	Satisfaction	4.15 (0.67)	4.36 (0.67)	4.17 (0.74)	0.0127*
Surgical treatment	Accuracy	4.25 (0.72)	4.35 (0.65)	4.46 (0.65)	0.0320*
	Comprehensiveness	4.06 (0.75)	4.22 (0.80)	4.25 (0.65)	0.0478*
	Conciseness	4.10 (0.79)	4.14 (0.69)	4.11 (0.74)	0.9739
	Satisfaction	4.17 (0.76)	4.28 (0.72)	4.33 (0.65)	0.1967
Total	Accuracy	4.24 (0.74)	4.33 (0.69)	4. 28 (0.72)	0.0800
	Comprehensiveness	4.01 (0.78)	4.17 (0.75)	4.08 (0.75)	0.0002***
	Conciseness	4.13 (0.73)	4.12 (0.77)	3.95 (0.79)	<0.0001****
	Satisfaction	4.20 (0.72)	4.28 (0.70)	4.18 (0.70)	0.0102*

^a^All statistics were described as mean (SD), while the *p* value was referred to as the Kruskal-Wallis test *p* value.

* refers to the *p* < 0.05, ** refers to the *p* < 0.01, *** refers to the *p* < 0.001, and **** refers to the *p* < 0.0001.

### 3.2 Quality evaluation in different content and difficulty levels

**Figure 2:**
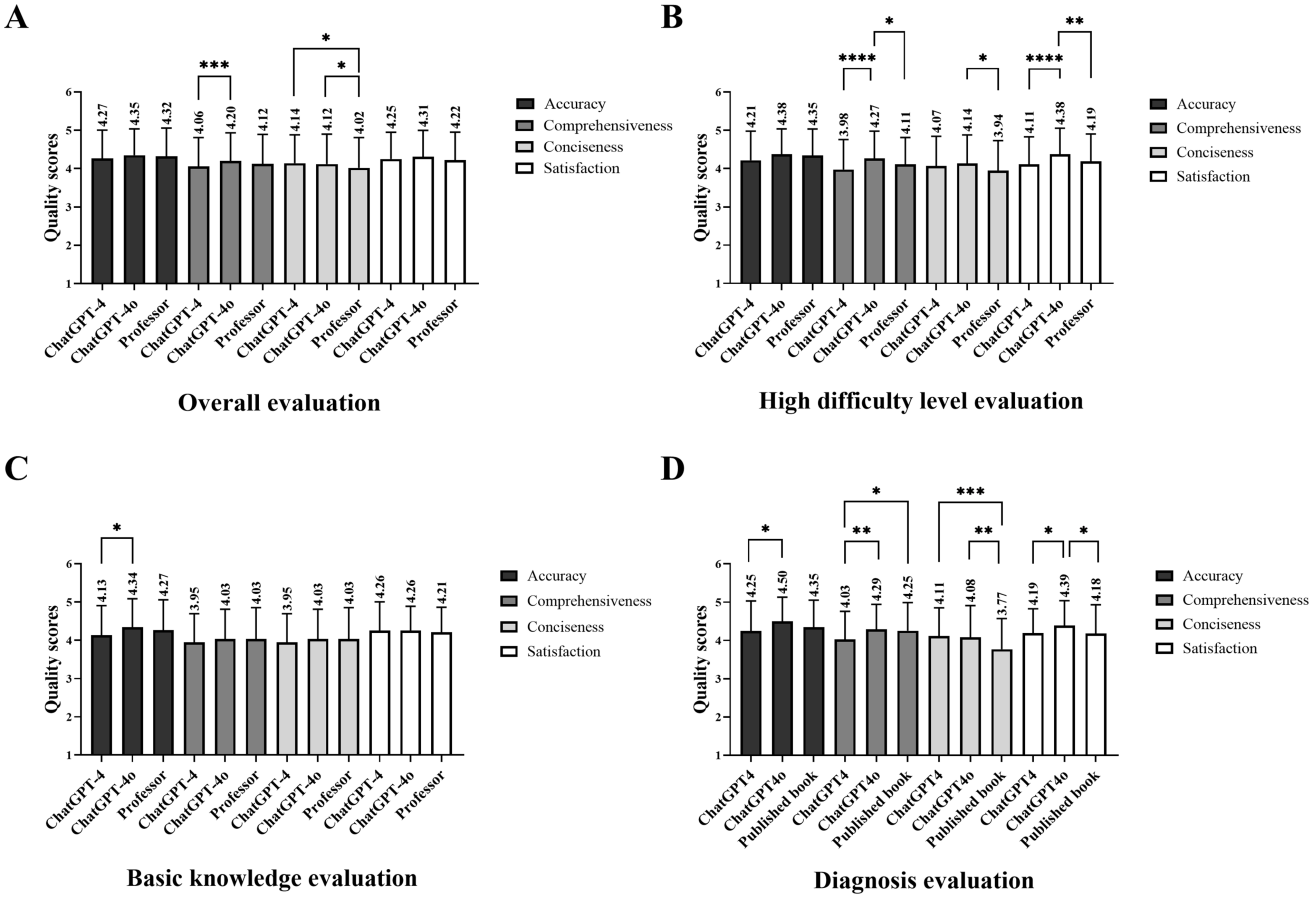
The performance of the LLM chatbot and the experienced ocular professor across quality dimensions. **(A)** Scores of overall quality evaluation. **(B)** Scores of quality evaluation on a high-difficulty level. **(C)** Scores of quality evaluation on diagnosis. **(D)** Scores of quality evaluation on surgical treatment (* refers to the *p* < 0.05, ** refers to the *p* < 0.01, and *** refers to the *p* < 0.001).

From the perspective of patients, ChatGPT-4o performed the best in accuracy, comprehensiveness, conciseness, and satisfaction. In detail, the mean accuracy scores of ChatGPT-4, 4o, and professor showed no significant differences, all rated 4 on the 5-point scale (4.24 [0.74], 4.33 [0.69], 4.29 [0.72]; *p* = 0.08). In particular, the mean performance of ChatGPT-4o in comprehensiveness amounted to 4, surpassing version 4 (4.17 [0.75], 4.01 [0.78]; *p* = 0.0001). The same was observed between 4o and the professor in terms of satisfaction (4.28 [0.70], 4.18 [0.70]; *p* = 0.0094) and conciseness (4.12 [0.77], 3.95 [0.79]; *p* < 0.0001, Table 2; Figure 2A). However, quality evaluation by ophthalmologists was not the same, showing no differences in all dimensions. An associate professor annotated the questionnaire in detail, highlighting some misinformation and briefly explaining several occasions (Table 5).

**Table 5:** The earnest annotations of the questionnaires from an associate professor.

Question (content, difficulty)	Answers	Annotate with comments
Question 1. Do all patients with hyperthyroidism occur with Thyroid Eye Disease? (Basic knowledge, low difficulty)	Ocular professor: Thyroid eye disease is often clinically manifested in two types: thyroid dysfunction ophthalmopathy (hyperthyroidism and hypothyroidism) and normal thyroid function ophthalmopathy. It is an autoimmune-related disease that is closely related to the immune response of the thyroid organ and is not directly related to hyperthyroidism. Thyroid eye disease occurs primarily in patients with Graves’ disease, which accounts for about 80% of all patients and has symptoms of hyperthyroidism. Another 10% of patients with Hashimoto’s thyroiditis (HT) have a more complex condition, showing hyperthyroidism, hypothyroidism, or normal thyroid function at different stages of their disease. In terms of time of onset, in about 10% of patients, thyroid-related eye disease occurs several years before thyroid disease. This condition is called euthyroid Graves’ disease. These patients do not have any signs of thyroid disease, but a complete sequence of thyroid antibody detection (thyroid peroxidase antibodies, thyroglobulin antibodies, blocking, stimulating, and binding antibodies to TSH receptors, and thyroid growth-stimulating immunoglobulins) reveals in almost all of these patients autoimmune evidence of the thyroid gland. Therefore, not all thyroid eye disease is associated with hyperthyroidism.	Do all patients with TED occur with hyperthyroidism? The answer is inconsistent with the question.
Question 4. What are the manifestations of Thyroid Eye Disease? Which one is the most characteristic and common? (Diagnosis, moderate difficulty)	ChatGPT-4o: Protrusion (exophthalmos): This is the most characteristic and common manifestation of thyroid-associated ophthalmopathy. Exophthalmos causes the eye to look more prominent than usual. Eyelid swelling: Eyelid swelling may affect the appearance. Diplopia: Seeing double shadows when looking at objects, usually due to the involvement of extraocular muscles, resulting in uncoordinated eye movement. Dry eyes or tears: Eyes feel dry, uncomfortable, or excessive tears. Eye pain or tenderness: Pain or tenderness around the eyes, especially during activity. Light sensitivity: sensitive to light and uncomfortable. Visual acuity changes: severe cases may lead to blurred vision or loss of vision. Eyelid retraction: The upper eyelid retracts, and the white eye is exposed more, giving a “surprise” expression. Conjunctival congestion: Congestion or redness of the white part of the eye. Among these symptoms, protrusion (exophthalmos) is the most characteristic and common manifestation. Exophthalmos not only significantly affects the appearance but may also be accompanied by other symptoms, such as dry eyes, diplopia, and light sensitivity. Therefore, exophthalmos is often the first symptom that patients and doctors notice. If any of the above symptoms occur, especially with hyperthyroidism, it is recommended to consult an ophthalmologist or endocrinologist as early as possible for professional diagnosis and treatment.	Summary: Exophthalmos and eyelid retraction are the most characteristic and common symptoms for patients with TED.
Question 14. I am a patient with Thyroid Eye Disease, but my thyroid function is not stable recently. Can I receive surgical treatment? (Surgical treatment, high difficulty)	ChatGPT-4: The treatment of Thyroid Eye Disease usually requires a combination of multiple factors, including the stability of thyroid function. If thyroid function is unstable, surgical treatment may bring additional risks or complications. Usually, clinicians recommend stabilizing thyroid function first, which may be managed with drug therapy or other non-surgical treatments. After the stabilization of thyroid function, surgical treatment may be considered if eye symptoms are severe or progressive. It is recommended to communicate in detail with the medical team to understand all feasible treatment options and their potential pros and cons. The specific suitability for surgery is best determined by a joint assessment between endocrinologists and ophthalmologists, who provide personalized treatment recommendations, taking into account overall health, thyroid function test results, and the specific symptoms and severity of the eye disease.	Side effects: epilepsy, possible exacerbation of the Condition: weakened therapeutic effect.

Regarding difficulty levels, ChatGPT-4o scored the highest and showed significant differences in comprehensiveness (low difficulty: *p* = 0.0126, moderate difficulty: *p* = 0.0387, high difficulty: *p* < 0.0001) and in high-difficulty level (accuracy: *p* = 0.0449, comprehensiveness: *p* < 0.0001, conciseness: *p* = 0.016, satisfaction: *p* < 0.0001). The accuracy scores of ChatGPT-4, 4o, and the ocular professor all amount to 4 (4.28 [0.74], 4.34 [0.71], and 4.34 [0.70], respectively). In terms of satisfaction and comprehensiveness, ChatGPT-4o’s performance exceeded the professor for low (*p* = 0.2082 and *p* = 0.0478, respectively) and high-difficulty questions (*p* = 0.0043 and *p* = 0.0236, respectively, Table 2; Figure 2B).

There were inter-group differences in content focuses, including basic knowledge of the disease, diagnosis, and surgical treatment. The mean accuracy score of the answers generated by ChatGPT-4o was 4, surpassing ChatGPT-4 in the diagnosis section (4.49 [0.62] vs. 4.25 [0.78]; *p* = 0.0144, Figure 2C), behind the professor in surgical treatment (4.35 [0.65] vs. 4.46 [0.65]; *p* = 0.028, Figure 2D). The mean performance of ChatGPT-4 in the diagnosis section was unacceptable in terms of comprehensiveness (4.02 [0.74]), worse than version 4o and professor (*p* = 0.008 and *p* = 0.0069, respectively). The mean conciseness scores of the professor lagged behind the chatbots (*p* both <0.0001), while differences in satisfaction scores were found between ChatGPT-4 and 4o (4.15 [0.67] vs. 4.36 [0.67]; *p* = 0.0168, Figure 2C).

### 3.3 Performance of ChatGPT in image-based preliminary diagnosis

In specific inquiries on several facial appearance images, ChatGPT-4 failed to make possible diagnoses in two cases (2/16), complaining of the lack of characteristic symptoms or a complete medical history to narrow the general scope. For the three possible differential diagnoses, ChatGPT-4 demonstrated 4 correct diagnoses among the 14 cases with specific diagnoses (28.57%; 95% CI: 8.39–58.10%), while ChatGPT-4o correctly diagnosed 5 cases as TED (31.25%; 95% CI: 11.02–58.66%), showing no significant difference. Based on the facial appearance and CT images provided, both ChatGPT-4 and 4o achieved preliminary diagnoses in all 16 cases (16/16). When combined with CT images from the horizontal and coronal positions, ChatGPT-4 and 4o demonstrated 31.25% accuracy (95% CI 11.02–58.66%) and 87.5% accuracy (95% CI 61.65–98.45%, *p* = 0.004), respectively.

### 3.4 Patients’ feedback on the questionnaire

Patients with TED have poor disease knowledge currently (13), partially because of deficiencies in high-quality patient consultation and education. In addition to the constant evolution of popularization, collecting patients’ evaluations and feedback is also necessary. The survey is a quality evaluation collection as well as a channel for acquiring knowledge and dispelling doubts about TED, and many patients take photographs for later learning. Furthermore, with expectations for disease popularization, 56 patients provided their opinions or advice, and the majority of them had positive comments. Astonishingly, a patient identified inappropriate information based on her great knowledge, while others concisely shared their personal experiences in diagnosis and treatment. However, several patients proposed a preference for treatment information, such as the approximate surgery cost, surgical indications, and complications, and a great need for professional explanation about eyelid retraction, the main reason for huge psychological pressure (14) (Supplementary eTable 5). These valuable suggestions offer important directions for effective consultation and disease popularization.

### 3.5 Spearman’s correlation coefficients for the variables and satisfaction

Satisfaction, which is an overall reflection of the evaluator’s attitude, is worth determining whether it correlates with response variables and other quality scores. We discovered significant correlations between satisfaction, accuracy, comprehensiveness, and conciseness (*r* = 0.3179, *r* = 0.3415, *r* = 0.399, and *p* all <0.0001, respectively). The sex and disease stages impacted the evaluation. In addition, in paragraphs and sections, the evaluators’ disease duration and knowledge levels were positively correlated with satisfaction scores (*r* = 0.0665, *p* = 0.0016; *r* = 0.0544, *p* = 0.009; *r* = 0.0502, *p* = 0.0173; *r* = 0.0550, and *p* = 0.0091, respectively, Table 6). From the perspective of ophthalmologists, satisfaction scores were positively correlated with accuracy, comprehensiveness, and conciseness (*r* = 0.5399, *p* < 0.0001; *r* = 0.6427, *p* < 0.0001; *r* = 0.2749, and *p* = 0.0002, respectively) and negatively correlated with the evaluator’s age and professional title (*r* = −0.2091, *p* = 0.0048; *r* = −0.2689, and *p* = 0.0003, respectively, Table 6). The dissimilar results revealed the potential biases between patients and ophthalmologists in the satisfaction evaluation.

**Table 6:** Spearman’s correlation analysis between response variables, other quality scores, and overall satisfaction.

Variables and analysis		Satisfaction from patients with TED	Satisfaction from professor
Response variables			
	Characters	*r* = 0.0232, *p* = 0.2704	*r* = 0.1348, *p* = 0.0712
	Paragraphs	*r* = 0.0665, *p* = 0.0016	*r* = 0.1379, *p* = 0.0649
	Sections	*r* = 0.0544, *p* = 0.009	*r* = 0.0882, *p* = 0.2389
	Images	*r* = −0.0176, *p* = 0.4052	*r* = 0.0893, *p* = 0.2334
Quality scores			
	Accuracy	*r* = 0.3179, *p* < 0.0001	*r* = 0.5399, *p* < 0.0001
	Comprehensiveness	*r* = 0.3415, *p* < 0.0001	*r* = 0.6427, *p* < 0.0001
	Conciseness	*r* = 0.3990, *p* < 0.0001	*r* = 0.2749, *p* = 0.0002

*r* refers to Spearman’s *r* vaules, while *p* refers to *p* (two-tailed).

## 4 Discussion

TED is an ocular disease related to endocrine disorders, and thousands of patients have consulted ophthalmologists and endocrinologists from nationwide hospitals but inevitably encountered difficulties during diagnosis and treatment. Due to the short inquiry time or inner anxiety emotions, sometimes they could not fully express their doubts and receive effective answers. In this case, almost every department delivers online educational health-disease materials to patients, enabling them to better understand their current conditions and assimilate treatment planning (15). In addition to the traditional Internet browsers and social media platforms, online AI-based tools like ChatGPT with encouraging performance on the simulated Medical Licensing Examination (11, 16, 17) and early detection and prediction of abnormalities (18–20) have gained increasing attention from patients seeking medical information.

This study serves as a foundation for assessing the practical application of LLM chatbots in a non-English language and promoting virtue healthcare innovation, especially in the ophthalmic field. Online, high-quality consultation and pre-diagnosis, in coping with clinical efforts, are fundamental in the effective patient-oriented management system. Abundant disease knowledge, identification of abnormal facial symptoms, especially eye appearances, and interpretation of imaging examination results are must-have skills for ophthalmologists as well as the key points in this study. We explored the capacity of the LLM chatbot (ChatGPT-4, 4o) to generate accurate, comprehensive, concise, and satisfactory responses to 15 frequently consulted questions and to conduct accurate preliminary diagnoses based on images in clinical practice. In combination with the feedback collection, these responses, along with answers provided by an experienced ocular professor, were compiled into three questionnaires presented in random order. After exclusion, 162 valid questionnaires completed by 150 patients with TED and 12 verified ophthalmologists were included for further analysis.

The image interpretation, a new feature, has extended ChatGPT’s capabilities beyond text (21, 22). The severity, course, and stage of the disease all have potential effects on the degree of exophthalmos and other symptoms in patients. In elderly patients, there were many wrinkles around the loose and swollen eyelids, which led to disease symptoms that were not clearly visible in images. The difficulty of image-based preliminary diagnosis varied significantly across different cases, which may cause selection bias. This should be discussed in further studies. For patients whose symptoms are not so obvious, the imaging examination results within the orbit are considered a supplement during TED diagnosis. Only based on several facial appearances and CT images, ChatGPT-4o diagonosed TED, displaying powerful capabilities of image information extraction and clinical matching, suppressing that of version 4. However, orbital cellulitis was the secondary preliminary diagnosis from ChatGPT, indicating a teaser deficiency in specialized information on the differential diagnosis of orbital lesions (23).

Every generator had its distinct features, and the LLM chatbot excelled in providing quantities of information, while the professor brought extensive clinical experiences into tailored advice. ChatGPT provided responses with a consistent structure: an introduction, point-by-point explanations, and a summary. There were several distinctive descriptions, including differences in indicator scope because of the equipment and the necessity to consult healthcare professionals for a personal plan. Compared with the cautious attitude in ChatGPT, the professor provided humanistic care in earnest.

With outstanding self-learning capacity, after some time, ChatGPT had significantly improved its responses to the same inquiries, especially on high-difficulty questions and image interpretation (24). We also observed notable advancements between versions (ChatGPT-4 and 4o), including amendments to translational errors inconsistent with language habits and improvements in comprehensiveness. Since long sentences and dense paragraphs make it difficult to maintain patience, it is necessary to separate into sections and use visual impacts such as bold fonts and website links, as in the logical and coherent responses from ChatGPT-4o. Therefore, unsatisfactory inquiries need to be repeated in further studies to verify self-learning and self-correct capabilities.

As seen in the feedback from patients, the majority of the patients expressed high satisfaction with the responses and proposed a preference for treatment information in further patient education, without identifying some of the answers generated by ChatGPT. Clinical decision-making recommendations are often considered weak spots of chatbots (16), classified as difficult-level questions in this study. ChatGPT-4o demonstrated prominence, much better than in previous research (25, 26), surpassing the professor in balancing accuracy, comprehensiveness, and conciseness. For example, in response to Question 11 (the timing of surgery), ChatGPT-4o pointed out that clinicians usually recommend surgery under stable conditions as listed in the advantages and disadvantages. These results highlight the potential benefits of key points for evolving patient consultation and disease management.

The overall satisfaction among patients demonstrated a positive correlation with scores on accuracy, comprehensiveness, and conciseness, and the four dimensions should be guaranteed in consultation and education. However, it remains challenging for LLM chatbot to keep impeccable with the avoidance of inaccurate, incomprehensive information all the time (27). In this study, without specific instructions, ChatGPT generated answers including some abbreviations to refer to the disease in the whole answer only after a brief introduction. However, the appearance of multiple terms (GD, Graves’ Disease; GO, Graves’ Ophthalmopathy; TAO, Thyroid Eye Disease and TED, Thyroid Eye Disease) in a single questionnaire increased the complexity for patients, leading to confusion. The reason may be that the pre-trained data for each response varied, and ChatGPT failed to distinguish various terms. In conclusion, although the performance of ChatGPT-4o was satisfactory for most circumstances, there’s still some distance from a perfect professor (11, 28).

Neither ChatGPT-4 nor 4o has the legal authority to provide diagnoses or treatment recommendations, under the guidelines of the American Academy of Ophthalmology (AAO) (29). To ensure the reliability and efficacy of eye care services assisted by chatbots, there’s a desperate need for targeted domain-specific pretraining under reliable databases (30). It is necessary to carry out supervision (29) that harnesses ChatGPT’s ability to tailor learning experiences (31) and ameliorate augmented learning (32). Healthcare professionals are supposed to address ethical concerns (33) and increase social acceptability (34) to enhance more beneficial integration of LLM chatbots into healthcare.

Due to the limited amount, the study included 15 common questions instead of specific questions about TED, whose bias may result in questions different from the essential topics to patients. Although some patients and ophthalmologists participated and left valuable feedback, including more TED experts, the latest disease guidelines, and widely recognized pathological views would have strengthened the quality comparison. Moreover, difficulty in distinguishing the exact quality was also a limitation, potentially leading to the reliance on self-criteria. Our findings on preliminary diagnosis were only based on several facial images and CT images from patients with TED, focusing on evaluating ChatGPT’s capacity to interpret images. Lacking information on patients’ medical histories, such as thyroid function indicators, specific symptoms, and underlying medical history, the occasion in this cross-sectional study may not be all the same in clinical practices, which should be considered in a more extensive study. The promising results of this cross-sectional study were specific to ChatGPT-4 and 4o and may not be generalized to other versions under special training. To apply Chatbot in clinical practices, its performance should be further assessed in the multicenter stimulation system.

In summary, ChatGPT-4o demonstrated satisfactory performance in generating responses to common clinical questions about TED and conducting accurate preliminary diagnoses based on images as a supplementary tool in the effective patient-oriented management system. Comprehensiveness and conciseness surpassing the ocular professor in decision-making questions. Although the performance of ChatGPT is promising in specific clinical scenarios, suppressing or approaching the experienced ocular professor, its recommendations should act as a reference under supervision.

## Data Availability

The original contributions presented in the study are included in the article/Supplementary material, further inquiries can be directed to the corresponding authors.
