# Supplementary material for: Evaluating the performance of ChatGPT in patient consultation and image-based preliminary diagnosis in thyroid eye disease
Source: Front Med (Lausanne). 2025 Feb 18;12:1546706. doi: 10.3389/fmed.2025.1546706 (PMC11876178; doi:10.3389/fmed.2025.1546706)
Supplement: Supplementary file 1 [file Table_1.docx]

Supplementary Material

**eTable 1.** Descriptions of 15 common clinical questions about Thyroid Eye Disease questions.

| Questions | Content categories | Difficulty level |
| --- | --- | --- |
| **Question 1.** Do all patients with hyperthyroidism occur with Thyroid Eye Disease?  **Question 2.** After all the treatment, will the patient’s orbit return to its previous state?  **Question 3.** Which people are prone to Thyroid Eye Disease? | Basic knowledge | Low difficulty  Moderate difficulty  Low difficulty |
| **Question 4.** What are the manifestations of Thyroid Eye Disease? Which one is the most characteristic and common?  **Question 5.** CT or MRI, what is more recommended for the imaging examination of patients with Thyroid Eye Disease?  **Question 6.** How do clinicians interpret the thyroid function checklist? | Diagnosis | Moderate difficulty  High difficulty  High difficulty |
| **Question 7.** How long does it take from the active stage to the stable stage during Thyroid Eye Disease?  **Question 8.** Why does Thyroid Eye Disease lead to exophthalmos in patients?  **Question 9.** Why does Thyroid Eye Disease lead to diplopia in patients? | Pathogenesis | Moderate difficulty  Low difficulty  Low difficulty |
| **Question 10.** How to choose the treatment time for patients with Thyroid Eye Disease?  **Question 11.** I am a patient with Thyroid Eye Disease, but my thyroid function is not stable recently. Can I receive surgical treatment?  **Question 12.** What is orbital decompression surgery and what are the containing surgical methods? | Surgical treatment | High difficulty  High difficulty  Moderate difficulty |
| **Question 13.** What is the latest medication progress for Thyroid Eye Disease? Is it possible to cure the disease without surgery?  **Question 14.** What are the precautions in the process of intravenous glucocorticoid therapy?  **Question 15.** What patients with Thyroid Eye Disease are suitable for radiotherapy? | Non-surgical treatment | Moderate difficulty  Low difficulty  High difficulty |

All fifteen questions were descriptive and designed to be answered from different aspects.

**eTable 2.** Description of the preliminary diagnoses from ChatGPT-4, ChatGPT-4o and the ocular professor.

| Cases | Images |  |  |  | Pre-diagnosis from ChatGPT-4 | Pre-diagnosis from ChatGPT-4o |
| --- | --- | --- | --- | --- | --- | --- |
| Case 1 | 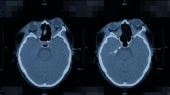 | 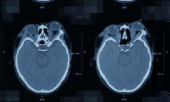 | 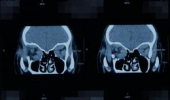 | 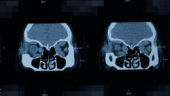 | Facial images:   1. Herpes Zoster Ophthalmicus 2. Bacterial Conjunctivitis with Cellulitis 3. Allergic Conjunctivitis | Facial images:  1. Blepharitis 2. Allergic Conjunctivitis 3. Herpes Simplex Keratitis |
|  |  |  |  |  | Facial+CT images:   1. Orbital Cellulitis 2. Orbital Pseudotumor (Idiopathic Orbital Inflammatory Syndrome) 3. Neoplastic Causes | Facial+CT images:  1. Orbital Cellulitis 2. Thyroid Eye Disease (Graves' Orbitopathy) 3. Orbital Tumor (e.g., Lymphoma) |
| Case 2 | 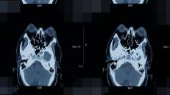 | 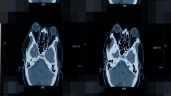 | 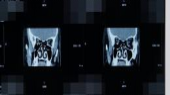 | 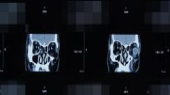 | Facial images:   1. Allergic Reaction 2. Thyroid Issues (like Hypothyroidism or Grave's Disease) 3. Cushing's Syndrome | Facial images:  1. Graves' Ophthalmopathy (Thyroid Eye Disease) 2. Orbital Tumor or Mass 3. Orbital Cellulitis or Other Orbital Inflammatory Conditions |
|  |  |  |  |  | Facial+CT images:   1. Sinusitis 2. Orbital cellulitis 3. Maxillary or orbital tumors | Facial+CT images:  1. Orbital Tumor (e.g., Meningioma or Orbital Pseudotumor) 2. Graves' Ophthalmopathy (Thyroid Eye Disease) 3. Orbital Cellulitis |
| Case 3 | 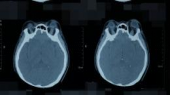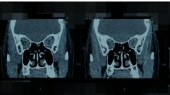 | 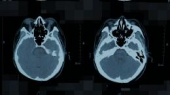 | 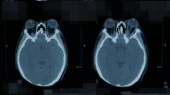 | 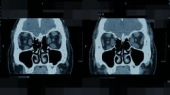 | Facial images:   1. Conjunctivitis 2. Keratitis 3. Uveitis | Facial images:  1. Graves' Ophthalmopathy (Thyroid Eye Disease) 2. Orbital Cellulitis 3. Orbital Pseudotumor |
|  |  |  |  |  | Facial+CT images:   1. Subconjunctival Hemorrhage 2. Conjunctivitis 3. Orbital Cellulitis | Facial+CT images:  1. Graves' Orbitopathy (Thyroid Eye Disease) 2. Orbital Cellulitis 3. Orbital Tumor |
| Case 4 | 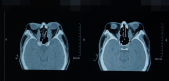  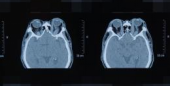 | 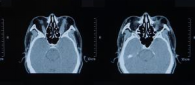 | 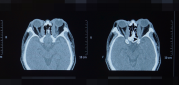 | 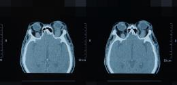 | Facial images:   1. Conjunctivitis (Bacterial or Viral) 2. Corneal Ulcer 3. Allergic Conjunctivitis | Facial images:  1. Orbital Cellulitis 2. Conjunctival Tumor or Mass (e.g., Conjunctival Squamous Cell Carcinoma) 3. Severe Allergic Conjunctivitis (Giant Papillary Conjunctivitis) |
|  |  |  |  |  | Facial+CT images:   1. Orbital Cellulitis 2. Allergic Conjunctivitis 3. Chemical Burn or Injury | Facial+CT images:  1. Orbital Cellulitis 2. Orbital Tumor (possibly lymphoma or a metastatic lesion) 3. Thyroid Eye Disease (Graves’ Orbitopathy) |
| Case 5 | 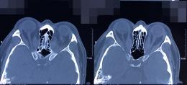 | 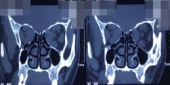 | 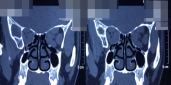 | 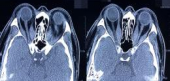 | Facial images:   1. Hypertension 2. Dermatological Issues 3. Allergic Reactions | Facial images:  1. Periorbital or Orbital Cellulitis 2. Allergic Reaction (Angioedema) 3. Sinusitis with Associated Facial Swelling |
|  |  |  |  |  | Facial+CT images:   1. Chronic Sinusitis 2. Nasal Polyps 3. Sinonasal Carcinoma | Facial+CT images:  1. Chronic Sinusitis with Orbital Complications 2. Orbital Cellulitis 3. Mucocele of the Sinuses |
| Case 6 | 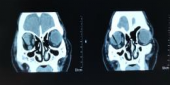 | 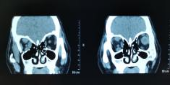 | 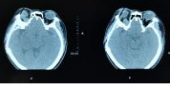 | 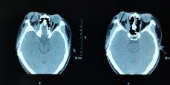 | Facial images:   1. Allergic Conjunctivitis 2. Blepharitis 3. Thyroid Eye Disease (Graves' Ophthalmopathy) | Facial images:  1. Oculomotor Nerve Palsy (Third Nerve Palsy) 2. Myasthenia Gravis 3. Horner’s Syndrome |
|  |  |  |  |  | Facial+CT images:   1. Orbital Cellulitis 2. Thyroid Eye Disease (Graves' Ophthalmopathy) 3. Sinusitis with Orbital Involvement | Facial+CT images:  1. Orbital Cellulitis 2. Thyroid Eye Disease (Graves' Orbitopathy) 3. Orbital Tumor (Lymphoma or Meningioma) |
| Case 7 |  |  |  |  | Facial images:   1. Conjunctivitis 2. Orbital Cellulitis 3. Thyroid Eye Disease (Graves' Ophthalmopathy) | Facial images:  1. Graves' Orbitopathy (Graves' Ophthalmopathy) 2. Orbital Cellulitis 3. Cavernous Sinus Thrombosis |
|  | 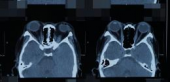 | 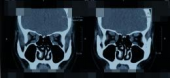 | 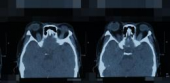 |  | Facial+CT images:   1. Orbital Cellulitis 2. Sinusitis with Orbital Involvement 3. Orbital Tumor | Facial+CT images:  1. Graves' Orbitopathy (Thyroid Eye Disease) 2. Orbital Tumor 3. Orbital cellulitis |
| Case 8 | 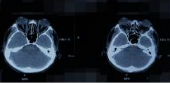 | 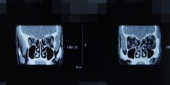 |  |  | Facial images:   1. Conjunctivitis 2. Episcleritis 3. Uveitis | Facial images:  1. Graves' Disease (Thyroid Eye Disease) 2. Orbital Cellulitis 3. Orbital Tumor (e.g., Meningioma or Lymphoma) |
|  |  |  |  |  | Facial+CT images:   1. Orbital Cellulitis 2. Thyroid Eye Disease (Graves' Ophthalmopathy) 3. Orbital Tumor | Facial+CT images:  1. Thyroid Eye Disease (TED) / Graves' Ophthalmopathy 2. Orbital Tumor (e.g., Lymphoma, Rhabdomyosarcoma) 3. Orbital Cellulitis |
| Case 9 | 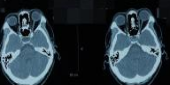 | 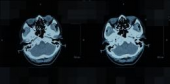 | 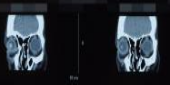 | 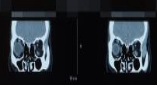 | Facial images:   1. Thyroid Eye Disease (Graves' Ophthalmopathy) 2. Allergic Reaction or Dermatitis 3. Bell's Palsy or another neurological disorder | Facial images:  1. Dermatochalasis 2. Blepharitis 3. Ptosis |
|  |  |  |  |  | Facial+CT images:   1. Sinusitis 2. Nasopharyngeal Carcinoma 3. Meningioma | Facial+CT images:   1. Orbital Cellulitis 2. Thyroid Eye Disease (Graves’ Ophthalmopathy) 3. Sinusitis with Orbital Extension |
| Case 10 | 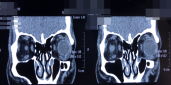 | 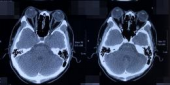 | 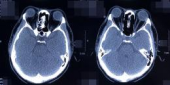 | 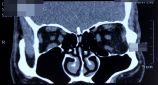 | Facial images:  NA | Facial images:  1. Bell's Palsy 2.Temporomandibular Joint Disorder (TMJ Disorder) 3. Trigeminal Neuralgia |
|  |  |  |  |  | Facial+CT images:   1. Brain Tumor (Primary or Metastatic) 2. Intracranial Hemorrhage 3. Cerebral Edema or Hydrocephalus | Facial+CT images:  1. Orbital Cellulitis 2. Sinus Mucocele or Mass (e.g., Ethmoid Mucocele) 3. Orbital Tumor (e.g., Lymphoma or Orbital Pseudotumor) |
| Case 11 | 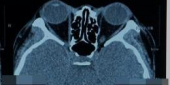  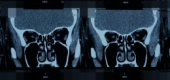 | 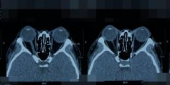 | 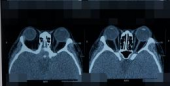 | 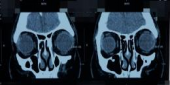 | Facial images:   1. Infection (like tuberculosis) 2. Malignancy (such as lymphoma) 3. Autoimmune Diseases (like rheumatoid arthritis or lupus) | Facial images:  1. Myasthenia Gravis 2. Horner's Syndrome 3. Third Nerve Palsy |
|  |  |  |  |  | Facial+CT images:   1. Chronic Sinusitis 2. Nasal Polyps 3. Sinonasal Tumors | Facial+CT images:  1. Orbital Tumor (likely a benign or malignant lesion) 2. Thyroid Eye Disease (Graves’ Orbitopathy) 3. Orbital Cellulitis or Abscess |
| Case 12 | 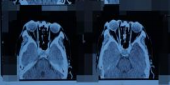 | 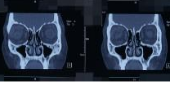 |  |  | Facial images:   1. Conjunctivitis 2. Blepharitis 3. Dermatitis | Facial images:  1. Blepharitis (Eyelid Inflammation) 2. Orbital Cellulitis 3. Allergic Conjunctivitis |
|  |  |  |  |  | Facial+CT images:   1. Orbital Cellulitis 2. Thyroid Eye Disease 3. Orbital Tumor | Facial+CT images:  1. Orbital Cellulitis 2. Thyroid Eye Disease (Graves' Orbitopathy) 3. Orbital Tumor (e.g., Lymphoma, Metastatic Disease) |
| Case 13 | 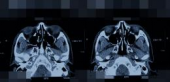  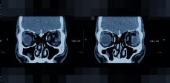 | 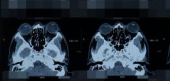 | 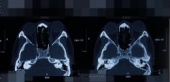 | 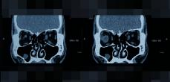 | Facial images:   1. Conjunctivitis 2. Uveitis 3. Glaucoma | Facial images:  1. Graves' Orbitopathy (Thyroid Eye Disease) 2. Orbital Tumor (e.g., Meningioma or Orbital Hemangioma) 3. Orbital Cellulitis (less likely in this case) |
|  |  |  |  |  | Facial+CT images:   1. Orbital Cellulitis 2. Thyroid Eye Disease (Graves' Ophthalmopathy) 3. Sinusitis with Orbital Involvement | Facial+CT images:  1. Graves’ Orbitopathy (Thyroid Eye Disease) 2. Orbital Tumor (e.g., Orbital Pseudotumor or Lymphoma) 3. Orbital Cellulitis |
| Case 14 | 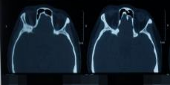 | 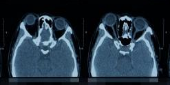 | 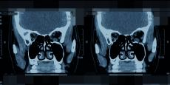 | 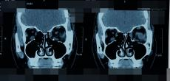 | Facial images:  NA | Facial images:  1. Periorbital Hyperpigmentation (Dark Circles) 2. Allergic Dermatitis 3. Mild Atopic Dermatitis |
|  |  |  |  |  | Facial+CT images:   1. Chronic Sinusitis 2. Nasal Polyps 3. Deviated Septum | Facial+CT images:  1. Thyroid Eye Disease (Graves' Orbitopathy) 2. Orbital Inflammatory Disease (Idiopathic Orbital Inflammation or Orbital Pseudotumor) 3. Orbital Tumors (e.g., Lymphoma or Rhabdomyosarcoma) |
| Case 15 | 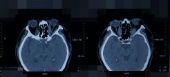 | 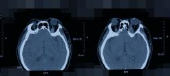 | 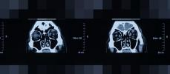 | 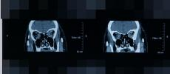 | Facial images:   1. Thyroid Eye Disease (Graves' Ophthalmopathy) 2. Orbital Cellulitis 3. Age-Related Changes | Facial images:  1. Allergic Conjunctivitis 2. Blepharitis 3. Orbital or Periorbital (Preseptal) Cellulitis |
|  |  |  |  |  | Facial+CT images:   1. Thyroid Eye Disease (Graves' Ophthalmopathy) 2. Orbital Cellulitis 3. Orbital Tumor | Facial+CT images:  1. Orbital Cellulitis 2. Thyroid Eye Disease (Graves' Orbitopathy) 3. Orbital Tumor (e.g., Lymphoma, Metastasis) |
| Case 16 | 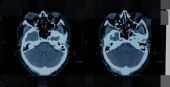 | 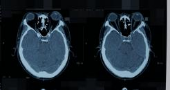 | 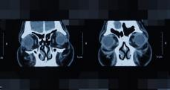 | 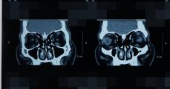 | Facial images:   1. Conjunctivitis (Pink Eye) 2. Corneal Ulcer 3. Episcleritis | Facial images:  1. Conjunctivitis (Pink Eye) 2. Subconjunctival Hemorrhage 3. Dry Eye Syndrome |
|  |  |  |  |  | Facial+CT images:   1. Orbital Cellulitis 2. Sinusitis with Orbital Complications 3. Neurological Conditions (e.g., Optic Neuritis, Tumor) | Facial+CT images:  1. Orbital Cellulitis 2. Sinusitis with Complications (e.g., Mucocele or Abscess) 3. Thyroid Eye Disease (Graves’ Ophthalmopathy) |

NA: Not applicable. All identifiable elements in the facial and CT images from patients with TED were mosaic processed.

**eTable 3.** Descriptions of the responses from ChatGPT-4, ChatGPT-4o, and the ocular professor.

| Questions (content, difficulty) | Responses from ChatGPT-4 | Responses from ChatGPT-4o | Responses from the ocular professor |
| --- | --- | --- | --- |
| **Question 1.** Do all patients with hyperthyroidism occur with Thyroid Eye Disease? (Basic knowledge, low difficulty) | Not all patients with hyperthyroidism occur with Thyroid Eye Disease, which is one of the most common complications of hyperthyroidism.  The occurrence of Thyroid Eye Disease may be related to the abnormal immune system of the patient. The immune system mistakenly attacks the eye tissue, leading to inflammation and tissue hyperplasia. However, even in the case of hyperthyroidism, not everyone will develop ocular lesions.  If you have hyperthyroidism and are worried that it may develop into Thyroid Eye Disease, it is recommended to discuss with clinicians for timely diagnosis and treatment. | In fact, only about 25-50% of patients with hyperthyroidism (Graves’ disease) develop varying degrees of thyroid eye disease (also called Thyroid-Associated Ophthalmopathy, TAO) symptoms. Factors affecting the occurrence and severity of TAO include:  **1. Smoking:** Smoking is an important risk factor for the development of TAO. The risk of TAO in smokers is significantly higher than that in non-smokers, and the symptoms are usually more severe.  **2. Gender:** Women are more likely to suffer from Graves’ disease and TAO, but when men develop TAO, the symptoms tend to be more severe.  **3. Age:** Middle-aged people ( 40-60 years old ) are more susceptible to TAO.  **4. Genetic factors:** Family history is also a potential risk factor.  **5. The degree and duration of thyroid dysfunction:** Inadequate treatment or long-term uncontrolled hyperthyroidism may increase the risk of TAO.  Although TAO is often associated with Graves’ disease, some patients may have TAO symptoms without obvious thyroid dysfunction. Therefore, although the two are closely related, not all patients with hyperthyroidism will develop TAO. | Thyroid eye disease is often clinically manifested in two types: thyroid dysfunction ophthalmopathy ( hyperthyroidism and hypothyroidism ) and normal thyroid function ophthalmopathy. It is an autoimmune-related disease that is closely related to the immune response of the thyroid organ, and is not directly related to hyperthyroidism.  Thyroid eye disease occurs primarily in patients with Graves’ disease, which accounts for about 80 % of all patients and have symptoms of hyperthyroidism. Another 10% of patients with Hashimoto’s thyroiditis (HT) have a more complex condition, showing hyperthyroidism, hypothyroidism, or normal thyroid function at different stages of their disease. In terms of time of onset, in about 10% of patients, thyroid-related eye disease occurs several years before thyroid disease. This condition is called euthyroid Graves’ disease. These patients do not have any signs of thyroid disease, but a complete sequence of thyroid antibody detection (thyroid peroxidase antibodies, thyroglobulin antibodies, blocking, stimulating, and binding antibodies to TSH receptors, and thyroid growth-stimulating immunoglobulins) reveals in almost all of these patients autoimmune evidence of the thyroid gland. Therefore, not all thyroid eye disease is associated with hyperthyroidism. |
| **Question 2.** After all the treatment, will the patient’s orbit return to its previous state? (Basic knowledge, moderate difficulty) | Thyroid Eye Disease ( TED ) is an autoimmune disease that can seriously affect the eyes and orbits. TED treatment aims to reduce inflammation, control thyroid function, and improve ocular symptoms.  After treatment, some symptoms may be significantly improved, such as eye pain, swelling, and dryness. However, some changes may be permanent, especially on the occasion of severe illness or delayed treatment. For example, exophthalmos or double vision may not fully return to its original state. In some cases, surgery may be required to rectify structural changes, such as ocular reattachment surgery or eyelid surgery.  In conclusion, the recovery degree of TED varies from person to person, depending on the severity of the disease, the timeliness of treatment, and the individual’s reaction to treatment. Timely cooperation between ophthalmologists and endocrinologists is the key to managing TED and getting back to the best possible condition, to develop an appropriate treatment plan based on the individual’s condition. | After treatment, whether the orbit can completely return to the original state depends on many factors, including the severity of the disease, the timeliness and efficacy of treatment, and the individual differences of patients.  **1. Severity of the disease:** Patients with mild thyroid eye disease may have a better recovery after treatment, and the orbital and ocular tissues may return to a near-normal state. In severe cases, even after adequate treatment, there may still be some permanent changes, such as orbital protrusion (exophthalmos), eyelid retraction and so on.  **2. Timeliness and efficacy of treatment:** Early detection and treatment can reduce eye damage and improve prognosis. A variety of treatment methods, including medication, radiation therapy and surgical treatment, may have better recovery if they can effectively control inflammation and reduce orbital pressure.  **3. Individual differences:** Each patient’s physical response and healing ability are different, which will also affect the recovery after treatment. Some people may have a full recovery, while others may have some long-term after effects.  In general, although many patients’ symptoms can be significantly improved after treatment, a full recovery to the original state is not a goal that everyone can achieve. For specific cases, it is best to consult a specialist who can provide personalized assessments and recommendations based on the specific circumstances of the disease. | After the orbital decompression surgery, strabismus surgery and eyelid retraction surgery are completed, various orbital symptoms may be alleviated, exophthalmos may be reduced, diplopia may be improved, and the appearance of the eye may be greatly improved, but it’s not possible to completely restore to the original state. In addition, since the course of thyroid eye disease may still be progressing, neither surgical treatment nor medication has changed the natural course of the disease, and the orbital symptoms may aggravate again. Therefore, after treatment, the orbit cannot be completely restored to the original state. |
| **Question 3.** Which people are prone to Thyroid Eye Disease? (Basic knowledge, low difficulty) | Thyroid Eye Disease ( TED ) is mainly related to hyperthyroidism, which is more common in the following types of people :  **1. Patients with hyperthyroidism:** TED is most common in Graves’ disease, an autoimmune disease leading to hyperactive thyroid.  **2. Smokers:** Smoking is an important risk factor for TED, which can increase the severity of the disease and the complexity of treatment.  3. Gender: Women are more likely to suffer from hyperthyroidism than men, and therefore are more likely to develop TED.  4. Genetic factors: People with a family history of thyroid disease are more likely to develop TED.  5. Age: Although TED can occur at any age, it is usually more common between 40 to 60-year-old people.  Knowing whether you are high-risk is helpful for timely monitoring and diagnosis. If you have eye problems such as exophthalmos, blurred vision, and high intraocular pressure, it’s supposed to consult healthcare professionals in time. | Thyroid-Associated Ophthalmopathy (TAO) is mainly associated with autoimmune thyroid diseases (especially Graves’ disease), but some people are more likely to suffer from this eye disease. The following are high-risk groups that are prone to TAO:  **1. People with Graves’ disease**  Graves’ disease: This is the most common related disease of TAO. Graves’ disease is an autoimmune hyperthyroidism in which the patient’s immune system mistakenly attacks the thyroid and orbital tissues.  **2. Smokers**  Smoking: Smoking is a major risk factor for TAO. Smoking not only increases the risk of disease, but also aggravates the symptoms of TAO and affects the therapeutic effect.  **3. Women**  Gender: Women are more likely to suffer from TAO than men. Although male prevalence is low, male patients are usually more severe.  **4. Middle-aged people**  Age: TAO is most common in middle-aged people, especially those between 40 and 60 years old.  **5. People with family history**  Family history: People with a family history of autoimmune diseases or thyroid diseases are more likely to suffer from TAO.This suggests that genetic factors may play a role in the pathogenesis of TAO.  **6. People with unstable thyroid function**  Thyroid function fluctuation: Patients with thyroid function not well controlled, frequent fluctuations or untreated are more likely to develop TAO.  **7. Patients with other autoimmune diseases**  Autoimmune diseases: People with other autoimmune diseases (such as rheumatoid arthritis, systemic lupus erythematosus, etc.) may be more likely to develop TAO.  **8. People with high stress**  Physical and physiological stress: Stress may trigger or aggravate the autoimmune response, thereby increasing the risk of TAO.  **9. People taking specific drugs**  Drug effects: Some drugs (such as iodine-containing drugs or anti-thyroid drugs) may affect thyroid function and indirectly increase the risk of TAO.  The incidence of TAO is mainly related to autoimmune response, and Graves’ disease is the main high-risk group. Smoking, gender, age, family history, thyroid function instability, other autoimmune diseases, stress status and the use of specific drugs are also risk factors for TAO. For high-risk populations, early screening and monitoring of thyroid function, as well as avoiding smoking and stress, can help prevent or mitigate TAO. | In theory, middle-aged, elderly and even young people, children may suffer from thyroid eye disease, and often in both eyes. At present, there is a lack of large sample size epidemiological survey data on thyroid eye disease worldwide. Indeed, some people are more likely to have thyroid eye disease than others. So, who is more susceptible to thyroid-related eye disease?  The risk of thyroid eye disease in patients with Graves’ disease is affected by several risk factors, including smoking, TSH receptor antibody, gender, hyperthyroidism treatment patterns, medications, aging and stress.  Smoking is the strongest risk factor for thyroid eye disease. People who have smoked all the time and exposed to second-hand smoke are more likely to suffer from thyroid eye disease. It is strong evidenced that there is a clear causal relationship between smoking and the development of thyroid eye disease. In 1987, Hagg and Asplund first confirmed the relationship between smoking and thyroid eye disease. Subsequently, a large number of studies have demonstrated that smoking is a risk factor for thyroid eye disease. Smoking increases the incidence of thyroid eye disease, increases its severity, reduces the response to treatment, and increases the probability of deterioration after radiotherapy. This is all clear. About 40 % of patients with thyroid eye disease have a history of smoking. In patients with thyroid eye disease, smokers are more likely to develop severe conditions, and the severity of the disease is closely related to the number of cigarettes smoked per day. Smokers with thyroid eye disease are more likely to have progression or insensitivity to treatment. The effects of medications for thyroid eye disease can be diminished by smoking. In patients with mild thyroid eye disease, radioiodine therapy induces disease development more frequently in smokers.  TSH receptor antibody (TRAb) may affect the course of thyroid eye disease. A study in a normal thyroid function population demonstrated that the activity and exophthalmos of thyroid eye disease are closely related to TRAb partial adhesion inhibitor antibody (TBII). The level of TBII in severe thyroid eye disease is higher. Therefore, TBII is an independent risk factor in addition to age and smoking.  Like hyperthyroidism, thyroid eye disease is more common in women. However, men are more common in patients with severe thyroid eye disease.  The Graves’ treatment pattern may also be a risk factor for thyroid eye disease. Subtotal thyroidectomy and antithyroid medications do not affect the course of thyroid eye disease. However, radioiodine therapy may induce the onset, development and even deterioration of thyroid eye disease.  The development of thyroid eye disease in patients with Graves’ disease may also be associated with susceptibility genes (e.g., HLA, CTLA-4, TNF, IFN-γ, CAM-1, TSH receptor). The results of these association studies with candidate genes have not been confirmed. In general, the incidence of thyroid eye disease is more dependent on environmental factors (especially smoking) than genetic factors.  Risk factors for thyroid-associated ophthalmopathy also include aging, stress, and medications such as lithium or interferon.  If sadness, anger, and aggravation are left unsaid and in the heart, these negative emotions will accumulate to create a significant amount of negative energy. When encountering major adverse events, such as the death of a loved one, spousal affair, relationship failure, loss of job, stock diving, etc., these negative energy will suddenly erupt, causing autoimmune disorders, leading to thyroid eye disease. In addition, disease. In addition, modern society is characterized by intense competition and increased work intensity and pressure, which can harm the body and mind, making it easy to suffer from thyroid-related eye diseases. |
| **Question 4.** What are the manifestations of Thyroid Eye Disease? Which one is the most characteristic and common? (Diagnosis, moderate difficulty) | The main manifestations of Thyroid Eye Disease (TED) include :  **1. Protrusion:** This is the most characteristic symptom of TED, resulting from the swelling of the tissue behind the eye (such as muscle and fat).  **2. Upper eyelid distraction:** This is a common symptom, the eyelids may be pulled backward abnormally, making the eyes look larger, and the whiteness of the eyes increases, especially in the upper eyelid.  **3. Eye discomfort:** including pain, burning sensation, dryness, itching, and feeling of a foreign body in the eye.  **4. Visual problems:** Patients may experience double vision or vision loss.  **5. Ocular muscle dysfunction:** The limited eye movement may lead to difficult focus and visual impairment.  **6. Eye swelling:** Eyelid and periocular skin may appear swelling.  Exophthalmos is the most characteristic symptom since it is rare in other diseases, and upper eyelid distraction is one of the most common symptoms. These symptoms not only affect the appearance and vision of patients but also may cause serious psychological and emotional issues. It’s recommended to consult an endocrinologist or ophthalmologist as soon as possible in case you suspect thyroid-related eye problems. | **Protrusion (exophthalmos):** This is the most characteristic and common manifestation of thyroid-associated ophthalmopathy. Exophthalmos causes the eye to look more prominent than usual.  **Eyelid swelling:** Eyelid swelling may affect the appearance.  Diplopia: Seeing double shadows when looking at objects, usually due to the involvement of extraocular muscles, resulting in uncoordinated eye movement.  **Dry eyes or tears:** Eyes feel dry, uncomfortable, or excessive tears.  Eye pain or tenderness: Pain or tenderness around the eyes, especially during activity.  **Light sensitivity:** sensitive to light and uncomfortable.  Visual acuity changes: severe cases may lead to blurred vision or loss of vision.  **Eyelid retraction:** The upper eyelid retracts, and the white eye is exposed more, giving a “surprise” expression.  **Conjunctival congestion:** Congestion or redness of the white part of the eye.  Among these symptoms, protrusion (exophthalmos) is the most characteristic and common manifestation. Exophthalmos not only significantly affects the appearance, but may also be accompanied by other symptoms, such as dry eyes, diplopia, and light sensitivity. Therefore, exophthalmos is often the first symptom that patients and doctors notice. If any of the above symptoms occur, especially with hyperthyroidism, it is recommended to consult an ophthalmologist or endocrinologist as early as possible for professional diagnosis and treatment. | The clinical manifestations of thyroid eye disease are complex and diverse, but rarely occur in one patient at the same time. One of the most characteristic and common signs is upper eyelid retraction, which affects 90-98% of patients.  In normal subjects, the upper eyelid margin covers the upper corneal margin by 1 to 2 mm in the vertical midline of the cornea, and the lower eyelid margin is tangent to the lower corneal margin. When looking down, the upper eyelid will droop as the eyeball turns down, always covering the upper edge of the cornea 1 ~ 2mm, with no upper or sclera exposure.  Forward gaze  Downward down  Upper lid recession, which is characteristic of thyroid eye disease, can be characterized by a flat stare with the upper lid positioned at or above the upper edge of the cornea, exposing the white sclera and giving the appearance of a “gleam”, commonly known as a “whitish gaze”. “Thyroid gaze” is a characteristic condition of widening of the lateral eyelid fissure, which is characterized by “staring”, i.e., the “peak of the eyelid moving out” sign.  Upper eyelid retraction in thyroid eye disease  The occurrence of upper eyelid retraction is multifactorial, including overactivity of levator muscle, scarring or restrictive factors, mechanical factors (such as exophthalmos), etc. In the early stage of thyroid eye disease, there may be a mild short-term upper eyelid retraction, which may be caused by excessive excitement of the autonomic nerve. With the progress of orbital disease, the levator and Müller muscles develop inflammatory reactions, and muscle overactivity can lead to upper eyelid retraction. In the late stage of thyroid eye disease, factors such as exophthalmos, fibrosis of eyelid muscles, and restrictive myopathy of the inferior rectus muscle can play a role in fueling and aggravating upper eyelid retraction.  When the white sclera can be seen underneath in a normal relaxed state, that is, the lower eyelid retraction. Lower eyelid retraction is generally caused by exophthalmos.  Lower eyelid retraction in thyroid eye disease  In thyroid-related eye disease, the upper eyelid recedes because a muscle in the upper eyelid ( Müller’s muscle) is involved. When the eyes look down, the upper eyelid of the affected side cannot be drooped with the downturned eyeballexposing the white of the eye (sclera) above the black eye (cornea), which is medically called “upper eyelid droop”. It affects the other muscle on the eyelid (levator muscle), and when the eyes open excessively, the white sclera above the cornea is exposed, called “upper eyelid retraction” and the blinking action is reduced. Some people can not close the upper and lower eyelids during sleep, exposing the white sclera below the cornea, and the patient often feels that the eyes are dry, a foreign-body sensation, photophobia and tearing, and mild vision loss.  Left eye upper eyelid tardiness  Left eye upper eyelid retraction  The eyelids of both eyes could not be completely closed (white sclera exposed ).  Thyroid eye disease has the following manifestations : 1) Eyelid retraction, the upper eyelid is more severe, and the retracted eyelid may be several millimeters higher than the upper edge of the cornea. One look big and one look small. The contour of upper eyelid retraction in thyroid eye disease is often manifested as lateral flare or eyelid peak shift sign, an appearance that is highly correlated with the pathological manifestations of thyroid eye disease and is its most common special symptom. 2) Monocular or binocular exophthalmos, with enlarged eyelid fissures and a “glazed” appearance. 3) When looking downward, the upper eyelid falls slowly or does not fall, which is called a positive delayed fall sign. 4) Extraocular muscle hypertrophy or fibrosis, eye movement disorder, double vision. 5) Eyelids edema, conjunctival congestion and edema, periorbital soft tissue swelling. 6) Decreased tear secretion and dry eyes. 7) The eyelids cannot be closed, especially during sleep. Long-term corneal exposure can lead to secondary exposure keratitis and corneal ulcer, which can lead to blindness if not treated in time. 8) Due to the larger eye, it is sometimes mistaken for ptosis of the other eye. 9) Elevated orbital and intraocular pressure, eye distension and pain, periorbital pain/headache, and sometimes accompanied by nausea.  When the eyeball moves vertically downward, the retracted upper eyelid is often delayed, that is, a late fall of the eyelid, with the eyelid remaining at a higher position. Other common signs include swelling of the periorbital tissue, conjunctival edema, congestion, and prominent eyebrow wrinkles.  Exophthalmos is also a common sign in patients with thyroid eye disease. The degree of exophthalmos in normal people is 12-14 mm, with a difference pf no more than 2 mm between the two eyes. The causes of exophthalmos mainly come from two aspects: the enlargement and thickening of extraocular muscles, and the deposition of a large amount of aminoglucosan ( mainly hyaluronic acid containing absorbable water ) in orbital tissue caused by inflammation, resulting in the increase of orbital adipose tissue. The enlarged extraocular muscles and increased orbital fat can only “move forward” due to the presence of bony inelastic orbits, which eventually leads to exophthalmos.  Exophthalmos is highly correlated with lower eyelid retraction. Patients with exophthalmos often show incomplete eyelid closure. Many of these patients, especially those with wide lid fissure, have spotted staining below the cornea under fluorescein. When the eye moves to one or more sides, most patients with thyroid eye disease show movement disorders of the eyeball, resulting in double vision, which is called “diplopia” in medicine. |
| **Question 5.** CT or MRI, what is more recommended for the imaging examination of patients with Thyroid Eye Disease? (Diagnosis, high difficulty) | In the option of CT or MRI, these two imaging techniques have their advantages and disadvantages :  **1. MRI:**  -MRI is excellent for displaying details of soft tissues, such as the muscles, fat, and other tissues behind the eyeball.  -MRI does not use radiation and is safer for patients who need multiple examinations.  MRI can more clearly present the inflammation, muscle hypertrophy, or compression after the eyeball.  **2. CT:**  The fast scanning CT suits for patients who cannot remain stationary in an MRI machine.  -CT is more effective in examining bone structures, such as orbital bones.  However, CT has radiation and its long-term health risk is slightly higher than that of MRI.  MRI is usually a better alternative for Thyroid Eye Disease because it provides more detailed soft tissue images, helping to assess inflammation and tissue changes around the eyes. For urgent or special circumstances, CT may be selected. However, it’s supposed to make the final examination choice based on the clinician’s assessment and take into account the patient’s specific conditions and the need for details. | The following is a comparison of the advantages and disadvantages of CT and MRI in the examination of thyroid eye disease:  **Orbital CT**  **Advantages:**  1. Skeletal clarity: CT images bones very clearly and is suitable for the evaluation of orbital skeletal structures and lesions, such as changes and fractures of the orbital bones.  2. Rapid: CT scanning is fast and suitable for the need to rapid image acquisition.  3. Widely available: CT equipment is more common and readily available in many hospitals and clinics.  4. High resolution: It can clearly show hypertrophy of the extraocular muscles and the detailed structure of the orbital contents.  **Disadvantages:**  1. Radiation exposure: CT uses X-rays and there is some radiation exposure, although usually at a low dose, but for patients requiring repeated examinations need to be carefully considered.  2. Limited soft tissue contrast: CT is not as good as MRI in soft tissue contrast and may not be as detailed as MRI for evaluating soft tissue lesions.  **Orbital MRI**  **Advantages:**  1. Excellent soft tissue imaging: MRI is superior to CT in terms of soft tissue contrast and is able to show soft tissue lesions more clearly in the extraocular muscles, optic nerves and the orbit.  2. No radiation: MRI does not use X-rays, so there is no radiation exposure, making it suitable for patients who require multiple imaging examinations.  3. Detailed Optic Nerve Evaluation: MRI can more clearly show the optic nerve and orbital inflammation, which is helpful to evaluate optic nerve compression.  4. Multiplanar imaging: MRI can acquire images in any plane, providing more comprehensive information about anatomical structures.  **Disadvantages:**  1. Longer examination time: MRI examinations usually take more time than CT and may not be suitable for rapid diagnosis.  2. Equipment limitations: MRI equipment is expensive and not available in all healthcare facilities, and some patients (e.g., those with metal implants or claustrophobia) may not be suitable for an MRI.  3. Higher cost: MRI is usually more expensive than CT.  Specific options  1. Initial evaluation: CT is usually the first choice for initial evaluation, especially for patients who need rapid identification of orbital structure and extraocular muscles.  2. Optic nerve compression: MRI may be more appropriate if optic nerve compression is suspected or a detailed assessment of soft tissue lesions in the orbit is required.  3. Repeat examinations: For patients requiring multiple follow-ups and examinations, MRI is more suitable due to no radiation exposure.  4. Physician’s recommendations: The final choice should be based on the patient’s specific situation and the physician’s recommendations, combined with the hospital’s equipment and the purpose of the examination, to choose the most suitable imaging examination.  CT and MRI have their own advantages and disadvantages, and the specific choice depends on the focus of the assessment and the patient’s specific circumstances. The physician will recommend the most suitable imaging examination based on the clinical manifestations, examination purposes and equipment availability. | CT and MRI have their own advantages and disadvantages, and can not replace each other.  Orbital CT horizontal and coronal scans showed different degrees of thickening of each extraocular muscle, and all 12 extraocular muscles can be observed. The horizontal scan showed an arc-shaped depression of the ethmoid bone paperboard into the ethmoid sinus, bilateral symmetry, showing “Cola bottle” sign. In patients with severe exophthalmos, the optic nerve is pulled straight and loses physiological curvature.  CT Coronal Scan Image  CT Horizontal Scan Image  Orbital MRI scan is similar to the CT scan, and the signal changes of the extraocular muscles correlated with the treatment. In the active stage of thyroid eye disease, the extraocular muscles showed inflammatory edema, with medium-low signal in T1-weighted and high signal in T2-weighted, suggesting that they were sensitive to hormone shock therapy and radiotherapy at this time. After extraocular muscle fibrosis, both T1 and T2 weighted signals were medium-low signal, suggesting that they were not sensitive to hormone shock therapy and radiotherapy at this time.  MRI Horizontal T1-weighted Image  MRI Horizontal T2-weighted Image |
| **Question 6.** How do clinicians interpret the thyroid function checklist? (Diagnosis, high difficulty) | The interpretation of the thyroid function checklist usually involves analyzing several key indicators, which can help determine whether your thyroid is working properly. Here are some common check indicators and their meanings:  **1. TSH (thyroid stimulating hormone):**  Normal range: about 0.4-4.0 mIU/L (different laboratories may vary slightly).  The pituitary gland in the brain secretes TSH to stimulate the thyroid gland to produce thyroid hormones. If the TSH level is high, it may indicate hypothyroidism, while if it is low, it may indicate hyperthyroidism.  **2. FT4 (free T4):**  Normal range: about 0.9-1.7 ng/dL (different laboratories standard may vary slightly).  FT4 is a kind of thyroid hormone, reflecting the activity of the thyroid. The low level of FT4 may indicate hypothyroidism, and the high level may indicate hyperthyroidism.  **3. FT3 (free T3):**  Normal range: about 2.0-4.4 pg/mL (different laboratories may vary slightly).  FT3 is also one of the thyroid hormones, and its change trend is similar to that of FT4, but FT3 reflects more on the body’s metabolic state.  **4. Antithyroid antibodies (such as anti-TPO antibodies and anti-TG antibodies):**  Detection of these antibodies can help determine the presence of autoimmune thyroid diseases, such as chronic thyroiditis or Graves’ disease.  When interpreting the thyroid function checklist, it is not necessary to focus on the absolute value of each indicator but also to combine the internal relationship, the individual’s symptoms, and health status. Professional clinicians should be the providers of final diagnosis and treatment. | To interpret the thyroid function checklist, it is necessary to understand the meaning and normal range of each indicator. The following are common thyroid function test indicators and their interpretations:  **1. Thyroid-stimulating hormone (TSH)**  -Function: TSH is secreted by the pituitary gland and regulates the production and release of thyroid hormones (T3 and T4).  -Normal range: usually 0.4-4.0 mIU/L (specific reference values may vary by laboratory).  -High TSH: It suggests hypothyroidism (hypothyroidism), that is, at low thyroid hormone level, and the pituitary gland increases TSH secretion to stimulate the thyroid gland.  -Low TSH: It suggests hyperthyroidism, that is, at high thyroid hormone level, and the pituitary decreases TSH secretion.  **2. Free thyroxine (FT4)**  -Function: FT4 is the main hormone secreted by the thyroid gland and participates in the regulation of metabolism in the body.  -Normal range: usually 0.8-1.8 ng/dL ( specific reference values may vary by laboratory ).  -High FT4: It suggests hyperthyroidism.  -Low FT4: It suggests hypothyroidism.  **3. Free triiodothyronine (FFT3)**  -Function: FT3 is the more active form of T4 after conversion in the body, mainly involved in metabolic processes.  -Normal range: usually 2.3-4.2 pg/mL ( specific reference values may vary by laboratory ).  -High FT3: It suggests hyperthyroidism.  -Low FT3: It suggests hypothyroidism, but FT3 decreased later.  **4. Total thyroxine (TT4) and total triiodothyronine (TT3)**  -Function: Includes protein-bound and free T4 and T3 in the blood.  -Normal range: TT4 is usually 5.0-12.0 μg/dL, TT3 is usually 80-180 ng/dL (specific reference values may vary by laboratory).  -The measurement of total T4 and total T3 can provide information on the total amount of thyroid hormones, but is greatly affected by serum protein levels.  **5. Thyroid autoantibodies**  -TRAb (TSH receptor antibody): mainly used for the diagnosis of Graves’ disease, high levels of antibodies suggest Graves’ disease.  -TPOAb (thyroid peroxidase antibody): common in Graves’ disease and Hashimoto’s thyroiditis, suggesting thyroid autoimmune diseases.  -TgAb (thyroglobulin antibody): It is also commonly used to evaluate thyroid autoimmune status.  **Interpretation examples**  1. Hyperthyroidism (Graves’ disease):  -TSH: below normal range.  -FT4 and FT3: above normal range.  -TRAb: positive, high levels suggest Graves disease.  2. Hypothyroidism (Hashimoto’s thyroiditis):  -TSH: above normal range.  -FT4 and FT3: below normal range.  -TPOAb and TgAb: positive, high levels suggest Hashimoto’s thyroiditis.  3. Subclinical hyperthyroidism:  -TSH: below normal range.  -FT4 and FT3: normal.  4. Subclinical hypothyroidism:  -TSH : above normal range.  -FT4 and FT3: normal.  **Specific steps of interpretation**  1. Examination of TSH levels: First look at TSH, as it is the most sensitive indicator.  2. Combination of FT4 and FT3: According to TSH abnormality, combined with FT4 and FT3 to further confirm hyperthyroidism or hypothyroidism.  3. Autoantibody detection: Auxiliary diagnosis of Graves’ disease or Hashimoto’s thyroiditis, to clarify the nature of thyroid disease.  Summary  The interpretation of the thyroid function checklist needs to combine the specific values with normal ranges of the indicators, considering the patient’s clinical symptoms and medical history. If there is any ambiguity, it is recommended to consult an endocrinologist for professional interpretation and advice. | In recent years, the prevalence of thyroid disorders has shown a significant upward trend. In many health examination items, thyroid function examination is also included. Faced with those high and low arrows on the thyroid function checklist, most patients are confused. So, what are the meanings of the high and low indicators on the thyroid function checklist ?  (1) Serum thyroid hormones (including TT3, TT4, FT3, FT4)  Thyroid hormone (TH) is an important indicator of thyroid function. It includes thyroxine (T4) and triiodothyronine (T3). Among them, T4 is all secreted by the thyroid gland, while only 20% of T3 comes from the thyroid gland, and the remaining 80% is transformed by T4 de-iodination in peripheral tissues. The physiological activity of T3 and T4 is different. The physiological activity of the former is 5 times that of the latter. The majority of T4 needs to be transformed into T3 to exert physiological effects.  Thyroid hormones have two forms: bound and free. The vast majority of them (more than 99 %) exist in the form of binding to plasma proteins (mainly thyroid-binding globulin, TBG), and the remaining small amounts of thyroid hormones are in the free state. Bound thyroid hormone is the storage and transport form of hormone, and free thyroid hormone (FT3, FT4) is the physiologically active form of hormone. Theoretically, free thyroid hormones are not affected by the change in serum TBG concentration and more truly reflect thyroid function.  The results of serum total T3 (TT3, abbreviated as T3) and total T4 (TT4, abbreviated as T4) are affected by serum thyroid-binding protein (TBG). When TBG increased (such as pregnancy, estrogen therapy, contraceptives, etc.), T3 and T4 increased; when TBG decreased (such as androgen and prednisone treatment, nephrotic syndrome, cirrhosis, etc.), T3 and T4 decreased. Therefore, free T4 (FT4) and free T3 (FT3) can truly represent the state of thyroid function. However, due to the low content of FT3 and FT4 in blood, the stability of the measurement results is not as good as that of T3 and T4. Therefore, FT3 and FT4 cannot completely replace TT3 and TT4 at present.  Generally speaking, the changes of FT3 and FT4 are consistent, increasing in hyperthyroidism and decreasing in hypothyroidism, but at a certain stage, the two may not be completely synchronized. For example, in hyperthyroidism, the increase of serum FT3 usually occurs earlier than the increase of FT4, so FT3 is more sensitive to the diagnosis of early hyperthyroidism and hyperthyroidism recurrence. When hypothyroidism occurs, it is often first manifested as a decrease in FT4, while FT3 can be normal (because TSH is elevated, it can promote the conversion of T4 to T3, so FT3 can be normal in patients with early hypothyroidism), so FT4 is more sensitive to the diagnosis of early hypothyroidism. In addition, “T3 hyperthyroidism” is mainly characterized by elevated FT3, FT4 may not increase; “T4 hyperthyroidism” is mainly characterized by an increase in FT4, and FT3 may not increase.  (2) Thyroid-stimulating hormone (TSH)  Thyroid stimulating hormone (TSH) is secreted by the pituitary gland, which has two main functions: stimulating thyroid gland to secrete thyroid hormone (TH), promoting the proliferation of thyroid tissue. TSH is regulated by the negative feedback of TH. When TH increases, TSH decreases, and when TH decreases, TSH increases. It should be noted that this “negative correlation” between the two is only applicable to primary hyperthyroidism or hypothyroidism, but not to pituitary hyperthyroidism or hypothyroidism, which is usually a “positive correlation”' relationship. For example, in patients with pituitary hyperthyroidism, because pituitary adenomas are autocrine and are not subject to feedback inhibition of thyroid hormones, they tend to have elevated FT3, FT4, and TSH; in patients with pituitary hypothyroidism, they tend to have lower TT4 and FT4, while TSH is decreased or not increased.  Thyroid stimulating hormone (TSH) is the most sensitive indicator of thyroid function. In the early stage of thyroid dysfunction, TSH often gives us “warning” before thyroid hormones (T3, T4), and if T4 and T3 begin to change, the change of TSH is often very significant. FT3 and FT4 are normal, TSH is reduced, known as “subclinical hyperthyroidism”', indicating that “hyperthyroidism” will occur. FT3 and FT4 are normal, TSH is elevated, known as “subclinical hypothyroidism”, indicating that “hypothyroidism” will occur.  (3) Anti-T3 (rT3)  Anti-T3 (rT3) is mainly formed by the deiodination of T4 during peripheral tissue metabolism. rT3 is similar to T3 in structure, but does not have physiological activity. T4 can be converted into T3 and rT3 in peripheral tissues. If the production of rT3 increases, the conversion of T4 to T3 decreases accordingly, which can reduce the consumption of oxygen and energy in the body. This is a protective mechanism for the organism. In hyperthyroidism, rT3 rises and falls in synchronization with T3, T4, FT3 and FT4, but it can also be elevated in the elderly, severely malnourished and patients with advanced malignant non-thyroidal diseases (e.g., “low T3 syndrome”), and is therefore less specific and less meaningful in the diagnosis of hyperthyroidism. In patients with “low T3 syndrome” (also known as “normal thyroid sick syndrome”, ESS), rT3 can be independently elevated when T3, T4, FT3, and FT4 are normal, but rT3 is decreased in patients with hypothyroidism, so rT3 is often used in hypothyroidism. Therefore, rT3 is often used to distinguish hypothyroidism from “low T3 syndrome”. The higher the ratio of rT3/TT3, the more serious the patient’s condition.  (4) Thyroid autoantibodies  Thyroid autoantibodies mainly include thyroid peroxidase antibody (TPOAb) and thyroglobulin antibody (TGAb). The measurement of serum thyroid autoantibodies is mainly used to identify the cause of hypothyroidism, and if the antibody level is significantly elevated, it is highly suggested to be “Hashimoto’s thyroiditis ( Hashimoto’s disease)”.  It should be noted that the level of thyroid autoantibodies is not directly related to the severity of thyroid disease, and it is usually not a target of clinical treatment. Therefore, it is not necessary to pay too much attention to the level of antibodies, and there is no effective drug to reduce antibodies.  (5) TSH receptor antibody (TRAb)  TSH receptor antibody (TRAb) actually contains two components: stimulatory antibody (TSAb) and inhibitory antibody (TSBAb). Among them, TSAb is a pathogenic antibody of Graves’ disease, and its positive plays an important role in the diagnosis and prognosis of Graves’ disease, and can also be used as a reference indicator for the discontinuation of antithyroid drugs (ATD). In addition, TSAb can also cause “neonatal hyperthyroidism” through the placenta, so it has a predictive effect on neonatal hyperthyroidism. However, due to the complex conditions of TSAb measurement, it has not been widely carried out in clinical practice. At present, TRAb positive is often regarded as TSAb positive. TSBAb plays an important role in the pathogenesis of hypothyroidism.  (6) Thyroglobulin (Tg)  Thyroglobulin (Tg) is secreted by thyroid follicular epithelial cells and stored in the lumen of the thyroid follicles. Under normal circumstances, a small amount of thyroglobulin can be released into the blood (<40μg/L). Thyroglobulin in patients with total thyroidectomy is often less than 5μg/L or even completely undetectable. If the serum Tg level rises again during follow-up, it indicates residual lesions or metastasis. Therefore, in clinical practice, the dynamic change of Tg is often used to observe the efficacy of surgery and to monitor whether differentiated thyroid cancer (DTC) recurs after surgery. If the blood Tg increases, it indicates tumor recurrence or metastasis. If it is reduced to undetectable, it indicates a good prognosis. It should be noted that the tumor tissue of medullary thyroid carcinoma originates from thyroid C cells, rather than thyroid follicular epithelial cells, so the serum Tg level of patients with medullary thyroid carcinoma is not increased or even decreased.  (7) Calcitonin (CT)  Calcitonin (CT) is a hormone secreted by thyroid parafollicular cells (C cells), and its main role is to regulate calcium and phosphorus metabolism. CT elevation is an important basis for the diagnosis of medullary thyroid carcinoma. |
| **Question 7.** How long does it take from the active stage to the stable stage during Thyroid Eye Disease? (Pathogenesis, moderate difficulty) | The transition time from active to stable Thyroid Eye Disease can vary from person to person. In general, the active phase usually lasts about 6 months to 2 years. During this phase, eye symptoms (such as redness, pain, tearing, and vision problems) may worsen or change. Subsequently, the condition enters what is known as a stabilization phase, when symptoms no longer deteriorate, but some may persist.  Management and therapeutic approaches play an important role in shortening the active phase and alleviating symptoms. Treatment usually includes the use of antithyroid medications, glucocorticoids, immunosuppressants, or biologics. In some cases, surgical treatment may also be required.  Therefore, the exact timing of progression and response to treatment for thyroid-related eye disease may vary from individual to individual. It is recommended to communicate regularly with doctors in order to adjust the treatment plan according to changes in the condition. | The time it takes for thyroid eye disease to progress from the active stage to the stable stage varies from person to person, usually ranges between 18 and 24 months. During the active stage, the patient’s eye symptoms, such as eyelid retraction, exophthalmos, and blurred vision, gradually worsen, while during the stable stage, these symptoms can stabilize and no longer worsen significantly. The specific time may be affected by the following factors:  **1. The severity of the disease:** The more severe the disease, the active stage may last longer.  **2. The timeliness and effectiveness of treatment:** Timely and effective treatment may shorten the duration of the active stage.  **3. Individual differences:** Each person’s physical condition and immune response are different, leading to different changes in the course of the disease.  The goal of treatment during the active stage is primarily to reduce inflammation and control symptoms, while treatment during the stable stage focuses on repairing and correcting the eye damage that has occurred. If you or a loved one is experiencing thyroid eye disease, it is recommended to consult a specialized ophthalmologist or endocrinologist for personalized treatment recommendations. | It is difficult to say how long it takes for a specific patient to progress from an active stage to a stable stage, but most patients take 6 months to 2 years. All patients with moderate to severe active stage need to receive radiotherapy or corticosteroid therapy, which can accelerate the progression from an active stage to a stable stage. |
| **Question 8.** Why does Thyroid Eye Disease lead to exophthalmos in patients? (Pathogenesis, low difficulty) | The main cause of exophthalmos in Thyroid Eye Disease is abnormal activity of the immune system, which mistakenly attacks the thyroid gland, the muscles and other tissues, leading to the following changes:  **1. Tissue edema and inflammation:** The immune response leads to inflammation and swelling of the ocular tissues, especially the muscle and adipose tissue in the posterior orbital region. This inflammation and swelling increases the volume and pressure within the orbit.  **2. Fibrosis:** Long-term inflammation may lead to tissue hardening and fibrosis, making the muscles in the orbit unable to stretch normally, thereby further increasing the pressure inside the orbit.  **3. Adipose tissue hyperplasia:** In some cases, there may be an increase in the number of orbital fat cells, which can also cause the eyeball to push forward and form exophthalmos.  These changes cause the eyeball to move forward (protrusion) and may trigger vision problems, eye pain, and appearance changes. Treatment usually requires the cooperation of endocrinologists and ophthalmologists and may include drug therapy, radiotherapy or surgical intervention. | Thyroid-Associated Ophthalmopathy (TAO), also known as thyroid eye disease. The main causes of exophthalmos are as follows:  **1. Increased orbital contents:** In patients with TAO, the orbital contents, including the extraocular muscles, fat, and connective tissues, are prone to inflammation and fibrosis. The swelling and hyperplasia of these tissues occupy more orbital space, causing the eyeball to be pushed forward.  **2. Inflammation and edema:** TAO is an immune-mediated inflammatory disease. The immune system attacks the tissues around the eye, leading to inflammation and edema in the extraocular muscles and other orbital tissues. This inflammatory response increases the volume of the orbital contents, thus pushing against the eyeball.  **3. Fibrosis and scarring:** As the disease progresses, the inflammatory response causes fibrosis, a hardening and thickening of the orbital tissue. This fibrotic process causes the extraocular muscles and other tissues to lose their elasticity, further pushing the eyeball, exophthalmos.  **4. Adipocyte hyperplasia:** In patients with TAO, orbital adipocytes also hyperplasia and hypertrophy, occupying more orbital space. This change likewise pushes on the eyeball and makes it protrude.  Exophthalmos is a prominent symptom of TAO, and patients often suffer from swollen eyelids, dry eyes, photophobia, blurred vision and other symptoms. In severe cases, exophthalmos may affect vision and even lead to optic nerve compression, causing loss of vision. Therefore, timely diagnosis and treatment of TAO is essential to protect the patient’s vision and eye health. | In patients with thyroid eye disease, 60% will have exophthalmos, which may be monocular or binocular. This axial protrusion of the eyeball is mostly mild or moderate in most cases, and severe in less than 5% of cases. The exophthalmos in patients with thyroid eye disease is due to autoimmune disorders. A large number of antigen-antibody complexes are deposited in the orbits, infiltrating soft tissues such as fat and extraocular muscles, resulting in an increase in the volume of orbital contents. The orbit is a cone structure surrounded by four bone walls, and the volume is fixed. Therefore, the orbital contents behind the eyeball will squeeze the eyeball forward, resulting in exophthalmos.  Orbital bone walls  Moreover, although relying solely on exophthalmos can not be diagnosed thyroid eye disease, however, it must be recognized that thyroid eye disease is the most common cause of unilateral and bilateral exophthalmos.  It should be reminded that the degree of exophthalmos cannot be directly related to the level of intraorbital pressure. Some patients has pronounced degree of exophthalmos, but the orbital pressure is still elastic. In other patients, the exophthalmos is less pronounced, but the posterior orbital pressure is high and the optic nerve was severely compressed. Patients with the latter type of thyroid eye disease are more likely to develop compressive optic neuropathy. |
| **Question 9.** Why does Thyroid Eye Disease lead to diplopia in patients? (Pathogenesis, low difficulty) | The causes of double vision in Thyroid Eye Disease are mainly related to the affected ocular muscles. The following are some main reasons :  **1. Eye muscle swelling and thickening:** Thyroid Eye Disease can cause inflammation of the muscles and tissues at the back of the eye, which in turn swells and thickens. This swelling can interfere with the normal movement of the eye muscles, making eye movements dysfunctional and difficult to coordinate.  **2. Eye protrusion:** Due to the swelling of the tissues at the back of the eye, the eye may protrude forward (exophthalmos), which also affects the function of the eye muscles and further prevents the vision focusing properly.  **3. Strabismus:** Impairment ocular muscle function can lead to strabismus, a problem with the alignment of eyes. Strabismus can cause both eyes to look at the same object at different angles, resulting in double vision.  **4. Adjustment disorder:** Swelling and functional limitations of the ocular muscles may also affect the eye’s ability to adjust the thickness of the lens to accommodate close and long-distance vision, which may also lead to blurred or double vision.  Treatment usually involves the use of drugs to control thyroid function, reduce eye inflammation, and surgery to correct eye muscle function or reduce exophthalmos when necessary. Additionally, wearing special glasses or using blackout lenses may also help patients reduce double vision. | Thyroid-Associated Ophthalmopathy (TAO) is an eye condition correlated with abnormal thyroid function. The main causes of double vision (diplopia) are as follows:  **1. Extraocular myopathy:** One of the most common features of TAO is inflammation and swelling of the extraocular muscles (the muscles that control eye movement). Inflammation and swelling can lead to fibrosis of the extraocular muscles, which restricts the normal movement of the eyeballs, resulting in the inability to move both eyes in a coordinated manner, resulting in diplopia.  **2. Exophthalmos:** As a result of the inflammation and swelling of the tissues, the pressure in the orbit increases and the eyeballs protrude forward (exophthalmos). In this case, the position of the eyeballs changes, affecting the visual alignment of both eyes, leading to diplopia.  **3. Increased orbital contents:** Patients with TAO have increased orbital contents (e.g., adipose tissue, muscle), which leads to limited eye movement and abnormal eye position, affecting visual alignment between both eyes.  **4. Muscle fibrosis:** As the disease progresses, fibrosis of the extraocular muscles can further limit the ability to move the eyeballs, making the eyes unable to synchronize in certain directions, leading to diplopia.  In the treatment of diplopia caused by TAO, a combination of medications (e.g., glucocorticoids), radiation therapy, and surgery (e.g., orbital decompression, extraocular muscle surgery) is usually needed to reduce inflammation, correct eye position, and improve extraocular muscle function. | There are 6 extraocular muscles attached to each eyeball, and their innervation nerves and muscles functions are normal so that these muscles can move in a balanced and coordinated manner, and the eyeball can be centered and rotate freely, resulting in binocular single vision without ghosting. The imbalance and incoordination caused by changes of muscle strength or elasticity of any extraocular muscles can cause double vision. However, patients with thyroid eye disease often have one or more extraocular muscles edema or fibrosis, and the muscle strength is not balanced. The eyes cannot guarantee to focus on the same object at the same time, and the image of each eye on the retina is different. Therefore, it is manifested as double vision, which is called diplopia in medicine. |
| **Question 10.** How to choose the treatment time for patients with Thyroid Eye Disease? (surgical treatment, high difficulty) | The timing of treatment for Thyroid Eye Disease usually depends on the activity and severity of the condition. Here are some guidelines for timing of treatment:  **1. Active phase of the disease:** During the active phase, the eye disease exhibits inflammation and tissue swelling. At this point, the main goals of treatment are to control inflammation, reduce symptoms, and prevent further tissue damage. Common treatments include the use of glucocorticoids (e.g., prednisone), immunosuppressive agents, or biologic agents (e.g., rituximab).  **2. Stable phase of the disease:** Once the disease has stabilized and the inflammatory symptoms have decreased, surgical treatment may be considered to correct anatomical changes caused by TED, such as exophthalmos (forward protrusion of the eyeball) and binocular line of sight inconsistency. The stable phase usually needs to last at least 6 months to ensure no further inflammatory activity.  **3. Other supportive treatments:** At any time, supportive measures can be taken, such as using artificial tears to alleviate dry eye symptoms, wearing sunglasses to protect the eyes, and taking a head-raising sleeping position to reduce eye edema.  Choosing the right timing for treatment requires close monitoring of changes in the condition and cooperation with ophthalmologists and endocrinologists to develop an individualized treatment plan. The patient’s overall health and specific symptoms are also important factors in decision-making when treating Thyroid Eye Disease. | The timing of treatment depends on the severity and activity of the disease and its impact on the patient’s quality of life. The following are general principles of treatment timing:  **1. Disease active stage:**  - Mild active stage: Conservative treatment is mainly adopted, such as local eye drops, artificial tears, eye masks and so on.  - Moderate to severe activity: Systemic immunosuppressive therapy, such as glucocorticoid therapy, is required. If the effect is not satisfactory, other immunosuppressants or radiotherapy may be considered.  **2. Disease stable stage:**  - Mild symptoms: Continue conservative treatment with regular follow-up observation.  - Moderate to severe symptoms: If there are severe visual impairment, exophthalmos, diplopia and other symptoms that affect the quality of life, surgical treatment such as orbital decompression surgery, eye muscle surgery or eyelid surgery may be considered.  **3. Emergency:**  - If optic nerve compression results in a dramatic loss of vision, immediate emergency treatment should be given, usually including high-dose glucocorticoid therapy and/or surgical decompression.  **Specific treatment steps**  **1. Conservative treatment:** For mild symptoms or early stages of the disease, the main measures include:  - Use of artificial tears to relieve dry eye symptoms.  - Wear sunglasses to protect the eyes from sand and sunlight.  - Use of eye masks at night to prevent dryness due to incomplete eyelid closure.  **2. Medication:**  - Glucocorticoid: Mainly used to control the inflammatory response in the active stage, either orally or intravenously.  - Immunosuppressant: For patients who are ineffective on glucocorticoid therapy, other immunosuppressants such as cyclophosphamide and cyclosporine may be considered.  **3. Radiation therapy:** For patients with moderately to severe active disease, especially those with poor response to medication, low-dose radiation therapy may be considered to reduce the number of inflammatory cells in the orbit.  **4. Surgical treatment:**  - Orbital decompression surgery: Primarily used to relieve severe exophthalmos or optic nerve compression.  - Ocular muscle surgery: Correction of diplopia and ocular muscle paralysis.  - Eyelid surgery: Improvement of inadequate eyelid closure and aesthetic problems.  The timing of treatment for thyroid eye disease depends on the activity and severity of the disease, taking into account symptoms and quality of life. Early intervention and reasonable treatment can effectively control disease progression and improve prognosis and quality of life. Regular follow-up and multidisciplinary collaboration are also an important part of managing the disease. | Once suffering from thyroid eye disease, the patient would like to be treated immediately and restore the original appearance. Due to the thyroid eye disease course of disease through two stages, early for the active stage, late for the stationary or stable stage, and patients find their eyes have different problems at different time, to the hospital treatment time also varies greatly, must according to the course of disease development stage, and the severity of the disease varies from person to person to choose the treatment.  Thyroid eye disease is a chronic disease with a great degree of uncertainty in its course. The main pathological changes in the active stage are extraocular muscles hypertrophy, orbital fat edema, resulting in eyelid edema, conjunctival congestion, edema, lacrimal caruncle edema, eyeball protrusion, etc. If the symptoms are obvious, it belongs to moderate degree or above, which seriously affects the patient’s life and work, and even the eyelid is incompletely closed, and the eyeball is exposed at night, resulting in corneal epithelial damage. Meanwhile, it is necessary to use corticosteroids, as with the mild disease, and use artificial tears to protect the cornea.  After six months to one year and a half of the active period, the patient gradually enters the stationary stage, with significant connective tissue hyperplasia and neovascularization in orbital fat and fiber . At this stage, the patient’s exophthalmos is stable, the eye congestion is not obvious, and the eye position is deviated and fixed due to muscle fibrosis. At this time, the use of hormones fail to regress the protruded eyeball and restore the eye position of strabismus, so the patient may undergo rehabilitation surgery.  There is a special case that requires early surgery when there is a risk of perforation of corneal ulcers and the optic nerve is compressed by the extraocular muscles, resulting in a progressive decline in vision. For patients in the stable stage, orbital decompression to correct the exophthalmos, extraocular muscle surgery to correct strabismus and diplopia, eyelid surgery to correct eyelid retraction, etc., which can be performed selectively. In order to avoid recurrence, thyroid function and eye condition are generally required to be stable for more than half a year before implementation. |
| **Question 11.** I am a patient with Thyroid Eye Disease, but my thyroid function is not stable recently. Can I receive surgical treatment? (Surgical treatment, high difficulty) | The treatment of Thyroid Eye Disease usually requires a combination of multiple factors, including the stability of thyroid function.  If thyroid function is unstable, surgical treatment may bring additional risks or complications. Usually, clinicians recommend stabilizing thyroid function first, which may be managed with drug therapy or other non-surgical treatments. After the stabilization of thyroid function, surgical treatment may be considered if eye symptoms are severe or progressive.  It is recommended to communicate in detail with the medical team to understand all feasible treatment options and their potential pros and cons. The specific suitability for surgery is best determined by a joint assessment between endocrinologists and ophthalmologists, who provide personalized treatment recommendations, taking into account overall health, thyroid function test results, and the specific symptoms and severity of the eye disease. | In general, it is recommended to perform surgery when thyroid function is relatively stable. This is because the instability in thyroid function may affect the outcome of surgery and the recovery process. The following are some considerations:  **1. Stability of thyroid function:** Surgery is usually recommended when thyroid function has reached a normal or relatively stable state. Functional instability may increase the risk of postoperative complications.  **2. Severity of eye disease:** If the eye disease has severely affected vision or quality of life and medication is not effective, the surgeon may weigh the risks and consider surgery.  **3. General health:** In addition to thyroid function, the surgeon will also assess your overall health, including the function of other vital organs such as the heart, liver and kidneys.  **4. Effectiveness of medication:** If you are receiving anti-thyroid medication or other related treatments, the physicians will assess the effectiveness of these medications and whether adjustments to your treatment regimen are needed.  It is recommended to consult professional specialists in endocrinology and ophthalmology for a detailed evaluation and discussion to develop a treatment plan that is best for you. | Generally speaking, thyroid function is an important indicator to distinguish the active or stable stage of thyroid eye disease. For patients with unstable thyroid function, we suggest that they should first go to the endocrinology department to control the thyroid function, so that it can be maintained at a relatively stable level for a period of time. At the same time, we should go to the ophthalmology department to control the exophthalmos. When the thyroid function is stable for more than half a year and the eye condition tends to be unchanged, the operation is safer and more effective than the unstable operation. It should be emphasized that FT3, FT4 are the most important indicators in the normal range, and TSH is close to normal, while TRab and TPOab are not necessarily required to return to normal levels.  It is reminded that the treatment should be carried out step by step and not be rushed. It is generally not recommended that high-dose Iodine131 without the protection of corticosteroids should be used for local radiotherapy of thyroid, so as to avoid the rapid decline of hyperthyroidism index, and exophthalmos may be significantly aggravated in the short term. |
| **Question 12.** What is orbital decompression surgery and what are the containing surgical methods? (Surgical treatment, moderate difficulty) | Orbital decompression is a surgical procedure mainly used to treat Thyroid Eye Disease. The purpose of the surgery is to reduce the pressure behind the eyes and improve the patient’s vision and eye appearance. The methods of orbital decompression surgery mainly include the following:  **1. Fat resection:** By removing the orbital adipose tissue to increase the orbital space, thus reducing the exophthalmos.  **2. Bone decompression:** Increase the orbital volume by removing part of the orbital bone. This approach may include removal of the lateral, bottom or top bones of the orbit.  **3. Combined decompression:** This approach combines fat resection and bone decompression to achieve more significant results.  The choice of procedure usually depends on the patient’s specific situation and the severity of the disease. Clinicians can develop a treatment plan based on the specific needs of patients and the risks and benefits of surgery. | Orbital decompression is a surgical procedure designed to relieve eye symptoms due to a variety of causes by reducing the pressure on the orbotal contents. The procedure is commonly used to treat exophthalmos (e.g., caused by thyroid eye disease), or increased orbital pressure due to tumors, trauma, or other causes. There are several ways to perform orbital decompression surgery, including the following methods:  **1. Bony decompression:**  - Lateral wall decompression: The volume of the orbit is increased by removing or thinning the lateral orbit bone. This method is commonly used for unilateral exophthalmos.  - Medial Wall Decompression: The intraorbital pressure is decreased by removing the medial orbital bone. It is often used in combination with other methods of decompression.  - Inferior wall decompression: The volume of the orbit is increased by removing bone from the bottom of the orbit. This method is usually used in severe bilateral herniation.  **2. Adipose Tissue Decompression:** The volume of orbital contents is decreased by excising or removing adipose tissue from the orbit, thereby decreasing intraocular pressure. It is usually performed on the basis of bone decompression surgery and is more effective.  **3. Combined decompression:** Combination of multiple decompression methods , such as simultaneous medial and inferior wall decompression, and adipose tissue decompression , may achieve a better therapeutic outcome.  The choice of orbital decompression surgery depends on the patient’s specific circumstances, including the degree of exophthalmos, the specific cause of orbital pressure, and overall health. Before surgery, the surgeon will conduct a detailed evaluation and develop a personalized surgical plan based on needs. | Any surgery that reduces the elevation of intraorbital pressure and its effects by enlarging the bony orbit and/or removing orbital fat is called orbital decompression. Orbital decompression surgery mainly includes two surgical procedures, orbital bone wall decompression surgery and orbital fat decompression surgery. Therefore, it is a misconception that orbital decompression means removing or grinding away bone.  The orbital bone wall decompression surgery is to achieve the purpose of decompression by removing the orbital bone wall and expanding the orbital volume. Depending on the way into the orbit, orbital bone wall decompression surgery can be divided into transnasal, transcutaneous, transconjunctival, translacrimal, endoscopic transnasal, or internal and external wall balanced decompression.Depending on the number of bone walls removed, it can be divided into one-wall decompression, two-wall decompression, three-wall decompression, or four-wall decompression.  Orbital bone wall decompression surgery is mainly aimed at three bone walls: the inner wall, lower wall and outer wall. Roughly estimated, each bone wall decompression can reduce the degree of exophthalmos by 2-3 mm. EUGOGOGO’s overall estimation is that the two-wall orbital decompression surgery can reduce the degree of exophthalmos by 3.2-4.8 mm (average 4.3 mm), and the three-wall orbital decompression surgery can reduce the degree of exophthalmos by 5.6-6.5 mm (average 6.0 mm).  Conventional bone wall area for orbital decompression surgery  a: outer wall decompression area, including conservative front (red), deep outer wall (green), expanded outer wall (blue); b: The inner and lower wall decompression area, including the inner wall (red) and the lower wall (yellow).  With each surgical modality, a balance between treatment outcome and side effects is considered. The traditional concept is that for moderate exophthalmos, orbital medial and inferior wall decompression surgery is still the most common procedure of choice; if it is a serious exophthalmos, it can be combined with orbital wall decompression. However, another diametrically opposed concept is that the preferred surgical procedure for orbital decompression surgery should be extraorbital or deep extraorbital wall decompression. Extraorbital decompression may not only meet the needs of the growing cosmetic population, but also has a low risk of surgery, including complications such as persistent diplopia and cerebrospinal fluid leakage.  Orbital fat decompression surgery, that is, by removing orbital fat tissue, reducing the volume of orbital tissue, to achieve the purpose of decompression.  The conventional area of orbital fat decompression surgery  Yellow: the suitable resection area, including the orbital fat tissue in the superior, medial, inferior and inferior outside ; blue : adipose tissue above the outer orbital, not suitable for resection ; Red line : the orbital approach of orbital fat resection.  Orbital fat decompression surgery, the orbital approach can be through the conjunctiva or through the lacrimal caruncle hidden incision, resection range can include the orbital upper, inner, inner and outer lower adipose tissue. Adipose tissue above the orbit, not only need to be cut through the skin incision can be removed, and can not achieve the purpose of orbital decompression, is not recommended! Rough estimation, each removal of orbital adipose tissue 1ml, can reduce the degree of exophthalmos 0.7mm. Overall, the total amount of orbital fat resection can reach 5.5-6.5 ml.  In general, orbital fat decompression surgery is a safe surgical procedure that not only improves exophthalmos, but also relieves high orbital pressure. Particularly, it’s suitable for patients with moderate exophthalmos caused by lipomatous thyroid eye disease, orbital fat prolapse, and facial disfigurement caused by venous congestion.  In recent years, orbital bone wall decompression combined with fat resection has been gradually popularized, which can not only increase the degree of regression of exophthalmos, but also reduce the risk of medically induced diplopia.  Patients are often concerned about whether orbital decompression surgery will leave scars? Generally speaking, if the orbital decompression is not performed through the skin approach, there will be no scar formation, and the scar of the eyelid fold (i.e., double eyelid) incision surgery is not obvious. |
| **Question 13.** What is the latest medication progress for Thyroid Eye Disease? Is it possible to cure the disease without surgery? (Non-surgical treatment, moderate difficulty) | In recent years, significant progress has been made in the treatment of Thyroid Eye Disease. In particular, the introduction of Teprotumumab (trade name Tepezza) is a non-surgical treatment option for TED. Teprotumumab is a humanized monoclonal antibody that reduces ocular inflammation and swelling by inhibiting the insulin-like growth factor-1 receptor (IGF-1R). According to FDA approval and clinical trial data, Teprotumumab has shown significant efficacy in reducing exophthalmos (ocular protrusion). After Teprotumumab treatment, the majority of patients in the treatment group experienced a reduction in exophthalmos of more than 2 millimeters, while the effect was relatively low in the control group.  Additionally, the study noted that common side effects of Teprotumumab include muscle cramps, nausea, hair loss, diarrhea, fatigue, hyperglycemia, hearing loss, dry skin, altered taste, and headache. Particular attention should be paid to the fact that pregnant women should not use this drug, and during treatment and within six months after treatment, women should use contraception to avoid pregnancy.  Overall, while Teprotumumab offers an effective non-surgical treatment option for TED patients, they should consult with clinicians in detail to assess the pros and cons of the treatment before using. The availability of this drug provides hope for TED patients who do not want or cannot undergo surgery. | In the last few years, there have been significant advances in the treatment of Thyroid Eye Disease (TED). In particular, the introduction of Teprotumumab (trade name Tepezza), is a non-surgical treatment option for TED. Teprotumumab is a humanized monoclonal antibody that reduces inflammation and swelling in the orbit by inhibiting the insulin-like growth factor-1 receptor (IGF-1R). Based on FDA approval and clinical trial data, Teprotumumab has been demonstrated significant efficacy in reducing ocular protrusion (exophthalmos). The majority of patients in the treatment group experienced a reduction in exophthalmos of more than 2 millimeters after Teprotumumab treatment, while the effect was relatively low in the control group.  Additionally, the study noted that common side effects of Teprotumumab include muscle cramps, nausea, hair loss, diarrhea, fatigue, hyperglycemia, hearing loss, dry skin, altered taste, and headache. Particular attention should be paid to the fact that pregnant women should not use this medication and that women should use contraception during and for six months after treatment to avoid pregnancy.  Overall, while Teprotumumab offers an effective non-surgical treatment option for TED patients, they should consult with their physicians in detail to assess the pros and cons of the treatment before using it. The emergence of this drug offers hope to TED patients who do not want or cannot undergo surgery. | On January 21st,2020, the US FDA approved a new drug Teprotumumab for the treatment of patients with moderate to severe active thyroid eye disease. This new drug is so effective with minimal side effects that it is likely to replace the traditional corticosteroid hormone with huge side effects and usher in a new erat.  Why does this drug have such an amazing effect? Teprumab is a human monoclonal antibody and an inhibitor of insulin growth factor-1 receptor (IGF-1R). We know that the two major autoantigens involved in the pathogenesis of thyroid eye disease are thyroid-stimulating hormone receptor (TSHR) and IGF-1R.These two antigens form a complex in structure and function. It can be said that the two are mutually “conspiracy” to participate in the pathogenesis of thyroid eye disease. When the IGF-1R blocker teprizumab was used to block the IGF-1R signaling pathway, it also blocked the TSHR signaling pathway, and teprizumab played a “double-edged sword” role.  The greatness of this new drug is that it can change the natural course of thyroid eye disease and prevent active stage from entering the stable stage. If the course of disease does not enter the stable phase, it is possible to cure thyroid eye disease without surgery, because most of the surgery is performed in the stable phase. Traditional corticosteroid therapy does not change the natural course of thyroid eye disease. At present, excellent experts in thyroid eye disease in China and abroad are conducting long-term observation and follow-up on patients with moderate to severe active thyroid eye disease who are treated with telbivudine. If the course of these active patients no longer enters the stable phase, then the non-surgical treatment of thyroid eye disease will be achieved. We believe that the day is around the corner. Bless the day come early! |
| **Question 14.** What are the precautions in the process of intravenous glucocorticoid therapy? (Non-surgical treatment, low difficulty) | When treating thyroid eye disease with hormone shock therapy, the following important matters need to be paid attention to:  **1. Close monitoring:** Thyroid function and eye conditions need to be regularly examined during treatment to monitor the therapeutic effect and any possible side effects.  **2. Side effect management:** Hormone therapy may cause a variety of side effects such as weight gain, increased blood glucose, decreased bone density, and heart problems. Physicians may recommend preventive measures such as dietary adjustments, exercise, and possible medication.  **3. Psychological support:** Thyroid eye disease may affect the patient’s appearance and vision, leading to psychological stress or self-esteem issues. It is very important to provide appropriate psychological support and counseling.  **4. Avoid smoking:** Smoking is a risk factor for thyroid eye disease, which can aggravate the condition. Patients should be advised to quit smoking.  **5. Use medications appropriately:** During the entire treatment process, medications must be used in strict accordance with the physician’s guidance. Excessive or irregular drug use may lead to serious health problems.  **6. Cooperate closely with the physicians:** Patients should report any unusual symptoms or concerns to the physicians in a timely manner, and any adjustments to their treatment plan should be discussed with the physicians.  Ensuring that these matters are properly addressed can help maximize the effectiveness of treatment and minimize potential negative effects. | Thyroid eye disease (Graves’ ophthalmopathy) requires the following precautions during hormone shock therapy:  **1. Regular monitoring:** Regular inspection of blood glucose, blood pressure, electrolyte levels, and electrocardiograms so that side effects can be detected and managed in a timely manner.  **2. Prevention of infection:** High doses of hormones can inhibit the immune system and increase the risk of infection. Special attention should be paid to personal hygiene and avoid contact with people with infectious diseases.  **3. Gastrointestinal protection:** Hormones have an stimulating effect on the gastrointestinal tract, which may lead to gastric ulcers or gastric bleeding. It is recommended to use gastrointestinal protective drugs such as proton pump inhibitors or H2 receptor antagonists.  **4. Prevention of osteoporosis:** Long-term use of hormones can lead to bone loss. Calcium and vitamin D should be supplemented, and bisphosphonates should be used if necessary.  **5. Mental health:** Hormones may cause mood swings, anxiety, insomnia and other mental symptoms. Attention should be paid to monitoring and timely communication with physicians.  **6. Dietary adjustments:** Pay attention to low-salt and low-sugar diet, avoid high-fat and high-calorie foods to minimize weight gain and metabolic disorders caused by hormones.  **7. Regular eye examination:** Hormone shock treatment may cause elevated eye pressure, regular intraocular pressure examination should be conducted to monitor the changes of ocular symptoms.  **8. Gradual reduction of dosage:** Avoid suddenly stopping the medication and gradually reduce the hormone dosage according to the physician’s instruction to prevent rebound of the disease and hormone withdrawal syndrome.  Strictly follow the physician’s instructions for treatment and follow-up, pay close attention to changes in body and timely report any discomfort or abnormalities. | Physicians and patients need to pay attention to observe the treatment effect and side effects, especially the manifestation of side effects. For example, gastric ulcer can cause stomach pain and lead to coffee-colored stools. Hypokalemia leads to numbness and paralysis of hands and feet, which should be attached to great importance because of its endangerment of the lives of patients. Osteoporosis systemic bone pain, etc., need to be dealt with as soon as it is detected. |
| **Question 15.** What patients with Thyroid Eye Disease are suitable for radiotherapy? (Non-surgical treatment, high difficulty) | Thyroid Eye Disease (TED) mainly affects the tissues around the eye. In some cases, radiation therapy can be one of the treatment options. Cases suitable for radiation therapy usually have the following characteristics:  **1. Moderate to severe disease:** Mild TED usually prefers non-invasive treatment, such as corticosteroids. Moderate to severe cases, especially those who do not respond well to steroid therapy, may benefit from radiation therapy.  **2. Active phase disease:** The active phase (inflammatory phase) of TED is the stage where radiation therapy may be more effective. This is because radiation therapy can help reduce inflammation.  **3. Affected ocular muscles:** When TED mainly affects the ocular muscles, leading to problems such as exophthalmos or binocular asymmetry, radiation therapy may help to reduce enlarged eye muscles and improve symptoms.  **4. Patients without surgical indications or at higher risk of surgery:** For patients who are not suitable for surgery or at high risk of surgery, radiation therapy offers an alternative.  The specific indications and plans for radiation therapy should be formulated by an experienced healthcare professional based on individual condition. Clinicians usually evaluate the patient's overall health, the severity of the condition, and other possible treatment options before considering radiation therapy. | Radiotherapy is an option in the management of thyroid eye disease, and may be suitable especially in the following circumstances:  **1.Moderate to severe active eye disease:** Radiotherapy is suitable for moderate to severe active eye disease, especially when orbital inflammation and edema are evident, and drug treatment is not effective or tolerable.  **2. Ineffective medications:** Radiotherapy may be an effective alternative for patients who do not respond to treatment with glucocorticoids or other immunosuppressive agents.  **3.Rapid progression of the disease:** In some cases, if the disease progresses rapidly and leads to severe visual threat, radiation therapy can quickly control ocular inflammation and edema and prevent further visual impairment.  **4.Recurrent ophthalmopathy:** For patients with recurrent thyroid eye disease, radiotherapy can be used as a long-term strategy to control the disease.  **5.Combined with visual impairment:** Radiotherapy can help relieve stress and improve symptoms when edema of ocular muscles and soft tissues compresses the optic nerve, resulting in decreased vision or binocular diplopia symptoms.  The decision for radiotherapy needs to be made jointly by the  ophthalmologist and radiation oncologist after a detailed evaluation of the  Patient’s condition. It is necessary to closely monitor ocular and systemic reactions during treatment to ensure the safety and effectiveness of the  treatment. | Radiation therapy is suitable for patients with the following conditions: (1) patients with moderate-to-severe thyroid eye disease; (2) conservative treatment for more than 3 months without significant improvement of exophthalmos; (3) contraindications to hormone therapy or side effects; (4) patients with persistent progression of exophthalmos on conservative medication and a strong desire to treat the ophthalmopathy. |

**eTable 4.** Descriptions of questionnaires collecting personal information, quality scores and feedback (Questions 1 to 23).

| Questionnaire | Detailed instructions |
| --- | --- |
| **Overall instructions** | Dear friends, thank you for participating in this survey by questionnaire. Please confirm again that you have been informed and consent before filling it out. This questionnaire aims to collect comments for the common questions about the disease from patients with Thyroid Eye Disease, including a personal information collection section and 15 common questions related to Thyroid Eye Disease, accompanied by answers. Please note that there are no ‘right’ or ‘wrong’ answers; we value your honest feedback and encourage you to score the answers freely according to your thoughts, without feeling pressured. At the end of the questionnaire, there is an optional section to collect your opinions or advice, looking forward to valuable comments. We promise that every answer will be fully respected, while your privacy will be strictly protected. Undoubtedly, your participation will assist in promoting the development of TED-related knowledge popularization and benefiting future patients in diagnosis and treatment. Last but not least, thank you once again for your contribution. |
| **Personal information** | The following are questions about your personal information. Please choose the appropriate option based on your own situation. |
| 1. Your initial: [gap fill] | For example: Zhang San, fill in: ZS. This questionnaire will not reveal any personal privacy information, only used to distinguish participants filling the questionnaire, thank you for your cooperation! |
| 2. What is your gender? [single choice] | Female  Male |
| 3. What is your age group? [single choice] | 18 to 20 years old, 21 to 30 years old, 31 to 40 years old, 41 to 50 years old, and 51 to 60 years old. |
| 4. What stage of Thyroid Eye Disease are you currently in? [single choice] | Active  Stable stage |
| 5. How long have you been diagnosed with thyroid eye disease? [single choice] | half of a year, half of a year to a year, a year to three years, three to five years, and more than 5 years. |
| 6. What clinical treatments have you experienced before? [multiple choice] | The experience of surgical and non-surgical treatment on hyperthyroidism, intravenous glucocorticoid therapy, orbital radiotherapy, monoclonal antibody therapy, and the orbital surgery treatment. |
| 7. Do you know much about Thyroid Eye Disease? [single choice] | poor knowledge without any understanding of TED, inferior knowledge without any understanding except the definition of TED, moderated knowledge with understanding of TED common symptoms, basic knowledge with understanding of disease symptoms, development, and stage, and excellent knowledge with proficient understanding of the specific treatment plans. |
| **Quality evaluation items** | The following are 15 questions that patients with Thyroid Eye Diseases commonly ask during their clinical consultations. There is no standard answer. Please answer them truthfully and effectively based on your own knowledge. Thank you for your cooperation! |
| 8-22. Fifteen common clinical questions about TED (Question 1 to 15) | Accuracy: completely incorrect, incorrect, average correct and incorrect, almost correct with a little mistake, and completely correct. Comprehensiveness: quite incomplete, incomplete with few perspectives, average complete and incomplete, comprehensive with some perspectives, and completely comprehensive with diverse perspectives. Conciseness: completely unconcise [complex answers with numerous words], unconcise [difficult answers with many words], average concise and unconcise, concise [easy answers with some words], and completely concise [straightforward answers using a few words]. Satisfaction: completely unsatisfactory [useless for evaluators], not satisfactory [a little useful], average satisfactory and unsatisfactory, satisfactory [quite useful], and completely satisfactory [all useful]. |
| **Feedback** | The 15 common questions about Thyroid Eye Disease have been evaluated. You can fill in the box with your suggestions or advice when answering this questionnaire. Thank you again for your cooperation! |
| 23. Your suggestions or advice on this questionnaire. [fill gap] | It is a question designed as an optional section for advice giving of unprofessional participants. |

**eTable 5.** Patients’ advice and recommendations on the questionnaire.

| Positive comments | Advice on further TED popularization | Inner feelings |
| --- | --- | --- |
| Very useful and comprehensive tips! | Overall professional. But the interpretation of indicators still needs intuitive examples. | Popularization-seeking treatment- guidance-cognition-  claim-answer |
| The explanations are very detailed and comprehensive! | I found some problems such as not all active patients need radiotherapy or hormones, others are very helpful! | Medicine dosage is key, adjustment is technique, sleep is guarantee, mood is foundation. |
| The questionnaire is very good. | Clear, but there’s no further explanation after describing correlations between exophthalmos and eyelid retraction, and some repetitive explanations. | I hope that this disease will make a major breakthrough soon, and there will be not such patients in the world. |
| Detailed information about self-condition to receive a better therapeutic efficacy. | Hope to include more information about eyelid retraction. | I have known more about TED, and become more confident about my condition and medical conditions. |
| A better understanding of TED, the principles, and types of orbital decompression surgery. | I’m a patient with hyperthyroidism for 10 years, and the delayed treatment led to only wait for difficult surgery. Hope more knowledge popularization! | I know how to read the report and some of the surgical programs, leading to some confidence in the treatment. |
| I have know that there’re active, stationary, and stable phases and my current state. | Mostly comprehensive to have a basic understanding. Part of the terms need some efforts for ordinary people. | I hope that there will be medication to treat such a disease. |
| All the questions are very comprehensive and detailed, easy for patients to understand! | Would the disease worsen after hormone or radiation therapy? I hope to popularize about this a little bit more. | I realize that decompression surgery is a very good choice, and I will answer such questions for others. |
| Learn more about TED treatment. | If the cost, side effects and complications of the surgery can also be involved, it’s much better. | It’s too painful with TED, low self-esteem and confidence, difficult registration, and poor consultation. |
| Through answering the questions, I have learned a lot, its seriousness and various treatment, as well as some recovery measures. | I would like to know the complications of the surgery and whether the swelling of the eyes can be improved after the surgery, if not, can I have another surgery? | A very thorough explanation. Thanks very much for all your efforts, I have gained confidence in the recovery of my eyes! |
| Very professional. Although diagnosed as hyperthyroidism for many years, I learned that I could have surgery for my eyes only in two years. | I have already had orbital decompression, strabismus, and eyelid retraction surgeries. I hope to know some post-operative care knowledge, such as how long I can fly and touch the water, and useful medicines. | I feel that it is not easy for patients with TED, and I hope that my loved one’s eyes will recover soon. |
